# Conditional-prevalence correction for liability-scale analyses of covariate-adjusted binary GWAS

**DOI:** 10.1371/journal.pgen.1012348

**Published:** 2026-10-30

**Authors:** Siyi Chen

**Affiliations:** School of Public Health, LSU Health Sciences Center, New Orleans, Louisiana, United States of America; The University of Iowa, UNITED STATES OF AMERICA

## Abstract

Adjustment for heritable covariates in genome-wide association studies of disease can induce collider bias, creating spurious genetic associations. Following bias correction, interpretation on the liability scale, which represents continuous disease susceptibility, requires an appropriate conversion from log odds. We derive this conversion under a Gaussian liability model for small genetic effects. The direct effect, excluding pathways through the adjusted covariates, and the collider-bias component share a common conversion factor. We characterize how this factor depends on variation in disease risk across covariate values and extend the derivation to case-control sampling. In simulations, the proposed conversion reduced the effect-size inflation associated with scaling based solely on disease prevalence. These results link existing collider-bias corrections to estimation of direct liability effects and clarify the interpretation of covariate-adjusted associations in population and case-control studies.

## Author summary

Genetic studies of disease often account for risk factors such as body mass index. This adjustment can create a spurious association, called collider bias, which several methods aim to remove. Researchers also convert genetic associations to a scale of underlying disease susceptibility, or liability, so that their magnitudes can be compared across studies. The usual conversion uses the overall frequency of disease. We show why adjusted studies need more information: people with different values of the adjusted risk factors can have different disease risks, even within the same population. We derive a conversion that accounts for this variation and show how it combines with collider-bias correction. In population studies, the fitted association model provides the required factor directly. Studies that recruit cases and controls in fixed proportions require a conversion that also accounts for that sampling design. Our results provide a basis for interpreting adjusted genetic associations in units of disease liability.

## 1 Introduction

Genome-wide association studies (GWAS) of disease often adjust for heritable risk factors. For example, type 2 diabetes (T2D) studies may adjust for body mass index (BMI) to investigate genetic effects that operate through pathways other than BMI [1]. Such a direct effect differs from the total effect, which also includes pathways through BMI.

Adjustment also changes which individuals are compared. If a single-nucleotide polymorphism (SNP) and an unmeasured cause of T2D both affect BMI, people with the same BMI can differ in that unmeasured cause according to their genotype. The resulting association between the SNP and the unmeasured cause can distort the estimated genetic effect on T2D. This is collider bias [2–4]. It is distinct from removing the pathway through BMI: even a variant with no direct effect on T2D may acquire an association after adjustment.

Several methods estimate this bias from genetic association data. Dudbridge et al. proposed a regression-based correction for studies of disease prognosis that condition on disease incidence [5]. Slope-Hunter estimates the bias using groups of variants with different association patterns [6], and Cai et al. developed weighted-regression and instrumental-variable approaches [7]. Wang et al. extended correction to multiple adjusted covariates under a continuous-outcome model [8]. After this subtraction, an adjusted binary-GWAS association remains in log-odds units.

To interpret genetic effects on underlying disease susceptibility, researchers often use the liability scale. The liability-threshold model represents susceptibility as a continuous, normally distributed variable, with disease occurring above a threshold [9,10]. An effect on standardized liability is a change in susceptibility measured in population standard deviations. Transformations to this scale are established for disease heritability [11]. Earlier work also examined how covariate adjustment and case-control sampling affect association-test power [12]; liability-based tests have incorporated external information about disease risk across covariate values [13].

Here we ask how a covariate-adjusted log-odds association should be converted to a direct liability effect after collider bias has been removed. The usual conversion uses population prevalence, the overall frequency of disease. An adjusted analysis, however, compares individuals with the same covariate values. In a BMI-adjusted T2D study, those comparisons occur within BMI groups with different disease risks. Two populations with the same overall prevalence can have different variation in risk across BMI and therefore require different conversions. Removing collider bias from the association does not determine this change of scale.

We derive the relationship between an adjusted logistic-regression coefficient and the direct genetic effect on liability, including the collider bias induced by adjustment. Under a Gaussian liability model and small genetic effects, the direct effect and the collider contribution share the same conversion factor. We derive the fitted-logistic factor, a conditional-prevalence approximation based on prevalence and covariate-explained liability variance, and the corresponding factor under case-control sampling.

Scale errors also affect genetic variance, polygenic scores, and Mendelian randomization (MR), which uses genetic associations to estimate exposure-outcome effects [14–17]. We evaluate conversion in individual-level simulations, combine it with published bias estimators, and examine downstream MR estimates. Full derivations and extended analyses are in S1 Text.

## 2 Materials and methods

Fig 1 shows the causal model underlying the derivation.

**Fig 1:**
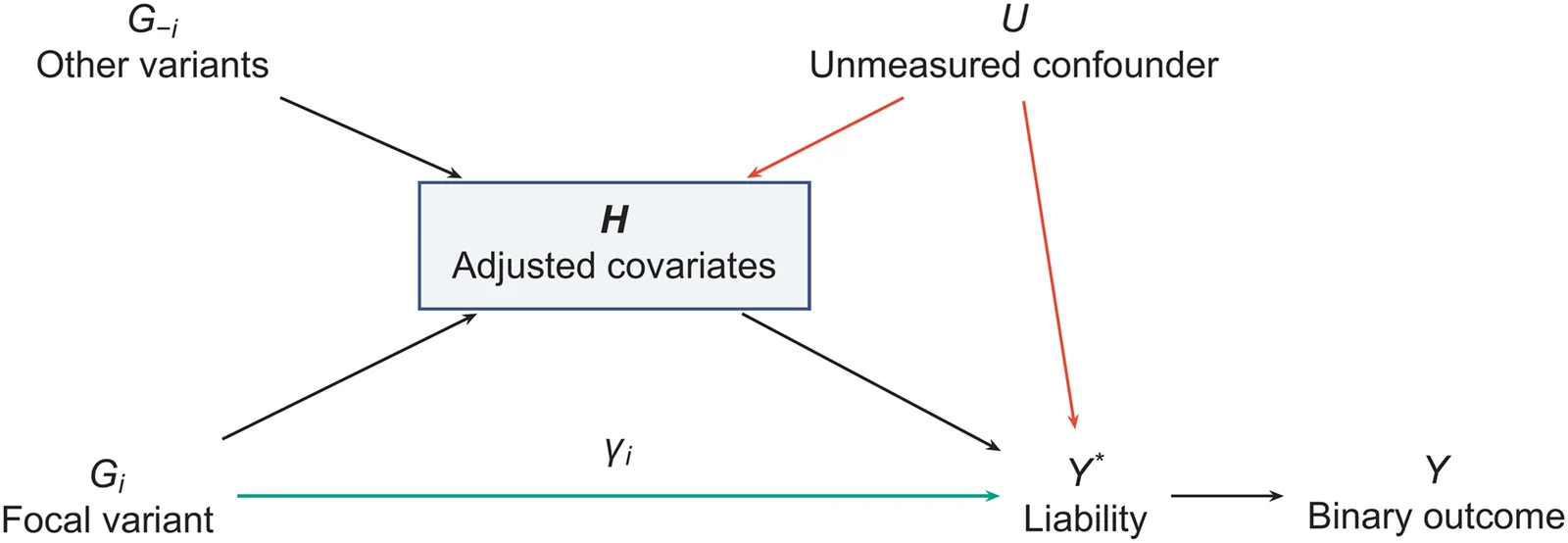
Hypothetical causal model. Adjustment for H blocks the mediated path and opens the collider path through *U*; $\gamma_{i}$ is the direct effect. Assumptions: Section 2.1.

### 2.1 Model and direct-effect target

Let $G_{i}\in \mathbb{R}$ denote a standardized genetic variant, Y∈{0,1} a binary disease outcome, $H\in {\mathbb{R}}^{q}$ a vector of adjusted heritable covariates, and J a vector of additional design covariates (age, sex, ancestry principal components, study site). A covariate-adjusted GWAS reports the logistic-regression coefficient ${\hat{\beta }}_{G_{i}Y}^{C}$ from the working model


$$\begin{array}{c} \text{logit}\mathrm{Pr}(Y=1|G_{i},H,J)=\alpha +\beta_{G_{i}Y}^{C}\;G_{i}+\delta_{H}^{⊤}H+\delta_{J}^{⊤}J. \end{array}$$ (1)

We work within a fixed design stratum and suppress J in the notation. In a pooled analysis, the conditional risk distribution includes all predictive design covariates, including those independent of $G_{i}$. Their associations with disease can change the liability-to-logit factor.

We adopt a liability-threshold model [9,10]. The disease outcome arises from an underlying continuous liability,


$$\begin{array}{c} Y^{*}=\gamma_{i}G_{i}+\delta_{H}^{*⊤}H+\alpha_{U}U+\eta ,\;\eta \sim 𝒩(0,\sigma_{\eta }^{2}),\;Y=1\{Y^{*}>t_{Y}\}, \end{array}$$ (2)

where *U* is a standardized unmeasured confounder of H and *Y*, $\delta_{H}^{*}$ are the partial liability-scale effects of H given $G_{i}$ and *U*, $\alpha_{U}$ captures direct effects of *U* on liability, and $\sigma_{\eta }^{2}$ is the variance of the residual liability noise. We standardize so that $\text{Var}(Y^{*})=1$, giving $t_{Y}=\Phi^{-1}(1-p_{Y})$. The covariates satisfy


$$\begin{array}{c} H=\beta_{G_{i}H}\;G_{i}+\alpha_{UH}U+\zeta , \end{array}$$ (3)

with ζ independent noise.

For the derivation, $G_{i},U,\zeta ,\eta$ are mutually independent and Gaussian, $\text{Var}(G_{i})=\text{Var}(U)=1$, and $\text{Var}(\zeta )=\Sigma_{\zeta }$ is positive definite. In particular, $G_{i}⟂U$. We use a Gaussian approximation to the focal genotype distribution for the local derivation. The end-to-end simulation in Section 3.5 uses binomial genotypes. Background effects of other variants on H enter ζ under the assumption that their aggregate is independent of the focal $G_{i}$ and *U*. Linkage disequilibrium with $G_{i}$ and genetic correlation with *U* are therefore relevant when assessing this model.

#### Target effect.

The target estimand is the liability-scale direct genetic effect $\gamma_{i}$. It differs from the total effect $\gamma_{i}^{tot}=\gamma_{i}+\delta_{H}^{*⊤}\beta_{G_{i}H}$, which includes mediation through H. The bias-subtracted coefficient targets the direct effect.

To obtain the adjusted association, write


$$\begin{array}{c} V_{0}=\alpha_{UH}\alpha_{UH}^{⊤}+\Sigma_{\zeta },\;c=V_{0}^{-1}\alpha_{UH}. \end{array}$$

Under joint normality,


$$\begin{array}{c} 𝔼[U|G_{i},H]=c^{⊤}\{H-\beta_{G_{i}H}G_{i}\}. \end{array}$$

Substitution into the liability equation gives its population partial regression on $\left(G_{i},H\right)$, the best linear prediction using these variables. The adjusted SNP coefficient is $\gamma_{i}+b_{lin}^{⊤}\beta_{G_{i}H}$, where $b_{lin}=-\alpha_{U}c$. The covariate coefficient is $\delta_{H}^{eff}=\delta_{H}^{*}+\alpha_{U}c$.

The covariates also determine the distribution of liability within the adjusted groups. At the focal-SNP null, $\left(\gamma_{i},\beta_{G_{i}H})=(0,0\right)$, define


$$\begin{array}{c} M(H)=\delta_{H}^{eff⊤}H,\;\rho^{2}=\text{Var}\{M(H)\},\;\tau =\sqrt{1-\rho^{2}}. \end{array}$$

Here τ is the conditional standard deviation (SD) of liability given H at the focal-SNP null. We take $0<p_{Y}<1$ and $0\leq \rho^{2}<1$. Joint normality makes the conditional variance constant, so the law of total variance gives $\tau^{2}=1-\rho^{2}$. It is generally distinct from the structural noise SD $\sigma_{\eta }$. In particular,


$$\begin{array}{c} \tau^{2}=\alpha_{U}^{2}\{1-\alpha_{UH}^{⊤}V_{0}^{-1}\alpha_{UH}\}+\sigma_{\eta }^{2}, \end{array}$$

which retains the part of the confounder unexplained by the measured covariates. Section S1.1 in S1 Text gives the complete conditional-mean and variance derivations.

### 2.2 Conditional liability-to-logit conversion

For an unadjusted (marginal) binary GWAS, a first-order Taylor expansion of the population logistic score equation under Eq (2) gives the standard result


$$\begin{array}{c} \beta^{logit,marg}\approx \lambda_{marg}\;\gamma_{i}^{tot},\;\lambda_{marg}=\frac{\phi (t_{Y})}{p_{Y}(1-p_{Y})}, \end{array}$$

which targets the total liability effect. After adjustment, let $p(H)=\Phi \{(M(H)-t_{Y})/\tau \}$ be the conditional probit risk at the focal-SNP null and $\dot{p}(H)=\tau^{-1}\phi \{(M(H)-t_{Y})/\tau \}$ its derivative with respect to a liability shift. Let $q_{0}(H)$ denote the fitted null working-logistic risk, with its intercept and covariate coefficients fitted jointly. The population logistic fit satisfies the joint score equation


$$\begin{array}{c} 𝔼\;[(Y-\sigma (\alpha +\beta_{G}G_{i}+\delta_{H}^{⊤}H))(1,G_{i},H^{⊤}{)}^{⊤}]=0, \end{array}$$

with σ(u)=expit(u). We expand at the focal-SNP null, where $G_{i}$ is independent of (Y,H) and its population fitted slope is zero.

The first-order population relationship is


$$\begin{array}{c} \beta_{G_{i}Y}^{C,*}=\lambda_{logit}(\gamma_{i}+b_{lin}^{⊤}\beta_{G_{i}H})+O(\epsilon_{i}^{2}), \end{array}$$ (4)

with


$$\begin{array}{c} \lambda_{logit}=\frac{{𝔼}_{H}\{\dot{p}(H)\}}{{𝔼}_{H}\{q_{0}(H)[1-q_{0}(H)]\}}, \end{array}$$ (5)

where $\epsilon_{i}=(\gamma_{i}^{2}+\|\beta_{G_{i}H}{\|}_{2}^{2}{)}^{1/2}$. Both the direct effect and the signed collider contribution shift conditional risk through the same derivative, and therefore share the same factor. The expansion holds as the focal effects approach zero, with the other structural coefficients fixed, positive conditional variance, and nonsingular logistic information. The $O(\epsilon_{i}^{2})$ remainder includes squared and cross-product terms. Section S1.2 in S1 Text gives the regularity conditions and joint score expansion, including the second-order adjustment that maintains unit liability variance.

At the focal-SNP null, the SNP information block is orthogonal to the intercept and covariate blocks. The null covariate score cancels the first-order contribution from the changing joint distribution of $\left(G_{i},H\right)$.

Replacing the fitted-logistic Bernoulli variance by the true conditional Bernoulli variance gives


$$\begin{array}{c} \lambda_{cond}=\frac{{𝔼}_{H}\{\dot{p}(H)\}}{{𝔼}_{H}\{p(H)[1-p(H)]\}}. \end{array}$$

The numerator is the average sensitivity of disease risk to a unit liability shift; the denominator is the average conditional Bernoulli variance.

Under the Gaussian model, $M\sim 𝒩(0,\rho^{2})$ and $𝔼[\dot{p}]=\phi (t_{Y})$. Gaussian convolution establishes this numerator identity; Jensen’s inequality gives $𝔼[p(1-p)]\leq p_{Y}(1-p_{Y})$. Consequently,


$$\begin{array}{c} \lambda_{cond}\;=\;\frac{\phi (t_{Y})}{{𝔼}_{M\sim 𝒩(0,\rho^{2})}\;[\Phi ((M-t_{Y})/\tau )\{1-\Phi ((M-t_{Y})/\tau )\}]}, \end{array}$$ (6)

$$\begin{array}{c} \kappa_{cond}=\frac{\lambda_{cond}}{\lambda_{marg}}\geq 1, \end{array}$$

with equality at $\rho^{2}=0$. The same lower bound holds for $\lambda_{logit}/\lambda_{marg}$ by the intercept score and Jensen’s inequality. Equation (6) is evaluated numerically by Gauss-Hermite quadrature. The proof and comparison of the two denominators are in Sections S1.4 and S1.5 in S1 Text; non-Gaussian covariates require separate risk modeling and prevalence calibration (Section S1.6 in S1 Text).

The denominator has a direct interpretation in terms of variation in conditional disease risk. By the law of total variance,


$$\begin{array}{c} 𝔼\{p(H)[1-p(H)]\}=p_{Y}(1-p_{Y})-\text{Var}\{p(H)\}. \end{array}$$

Under the Gaussian numerator identity, the approximate scale ratio can therefore be written as


$$\begin{array}{c} \kappa_{cond}={\left[1-\frac{\text{Var}\{p(H)\}}{p_{Y}(1-p_{Y})}\right]}^{-1}. \end{array}$$

Greater variation in conditional risk reduces the average Bernoulli variance and increases the factor. Here Var{p(H)} is variation on the probability scale, whereas $\rho^{2}$ is explained variance on the liability scale.

### 2.3 Combining conversion with collider-bias subtraction

Equation (4) gives the population bias slope $b_{logOR}=\lambda_{logit}b_{lin}$. We estimate it in adjusted log-odds units under the identifying and sampling assumptions of the chosen method [6,8,18] (Section S3 in S1 Text).

The bias-subtracted coefficient


$$\begin{array}{c} A_{i}\equiv {\hat{\beta }}_{G_{i}Y}^{C}-{\hat{b}}_{logOR}^{⊤}{\hat{\beta }}_{G_{i}H} \end{array}$$

converges in probability to $A_{i}^{*}=\lambda_{logit}\gamma_{i}+O(\epsilon_{i}^{2})$ when the adjusted GWAS coefficient, bias slope, and SNP-covariate associations are consistently estimated. Bias subtraction removes the collider contribution on the log-odds scale; the remaining operation is liability-scale conversion:


$$\begin{array}{c} {\hat{\beta }}_{G_{i}Y^{*}}^{cond}\;\equiv \;\frac{A_{i}}{{\hat{\lambda }}_{cond}}\;=\;\frac{A_{i}}{{\hat{\lambda }}_{marg}\;{\hat{\kappa }}_{cond}}\;=\;\frac{{\hat{\beta }}_{G_{i}Y^{*}}^{marg}}{{\hat{\kappa }}_{cond}}, \end{array}$$ (7)

Here ${\hat{\beta }}_{G_{i}Y^{*}}^{marg}=A_{i}/{\hat{\lambda }}_{marg}$ divides the same bias-subtracted numerator by the marginal factor. We refer to the two estimators as conditional-scaled and marginal-scaled. With known or consistently estimated factors, the conditional-scaled estimator converges in probability to $\left(\lambda_{logit}/\lambda_{cond})\gamma_{i}+O(\epsilon_{i}^{2}\right)$ and the marginal-scaled estimator to $\left(\lambda_{logit}/\lambda_{marg})\gamma_{i}+O(\epsilon_{i}^{2}\right)$. Dividing by $\lambda_{logit}$ removes this approximation error and leaves only the local remainder.

For empirical bias recovery, the across-SNP decomposition is


$$\begin{array}{c} \beta_{G_{i}Y}^{C,*}=b_{logOR}^{⊤}\beta_{G_{i}H}+c_{i}, \end{array}$$

where $c_{i}$ includes the direct contribution $\lambda_{logit}\gamma_{i}$ and higher-order terms. Regression across variants identifies the slope when the direct effects are uncorrelated with the SNP-covariate associations and measurement error in those associations is corrected, as in the corrected weighted least squares (CWLS) estimator of Cai et al. [7], implemented in the indexevent framework [5]. Clustering and robust MR methods use alternative assumptions, described in Section S3 in S1 Text.

### 2.4 Calibration, sampling, and uncertainty

#### Calibration inputs.

The Gaussian conversion uses population prevalence $p_{Y}$ and the observational covariate-explained liability variance $\rho^{2}$. For multiple adjusted covariates, $\rho^{2}=\delta_{H}^{eff⊤}\Sigma_{H}\delta_{H}^{eff}$ at the null, where $\Sigma_{H}$ is their population covariance matrix. This variance includes genetic and environmental associations with liability. In a population sample, the GWAS model supplies the working-logistic factor without a separate estimate of $\rho^{2}$. Under Gaussian covariate risk, the numerator of Eq (5) is $\phi (t_{Y})$ and its denominator is the average fitted Bernoulli variance of the null logistic model, so that


$$\begin{array}{c} {\hat{\lambda }}_{logit}=\frac{\phi ({\hat{t}}_{Y})}{n^{-1}\sum_{j=1}^{n}{\hat{q}}_{j}(1-{\hat{q}}_{j})}, \end{array}$$

where ${\hat{q}}_{j}$ is the fitted risk of participant *j*. Section S6.3 in S1 Text evaluates this estimate.

A representative individual-level cohort provides the covariate-risk relationship and joint covariate distribution for calibration. If a population probit model has predictor $u^{⊤}H$ with variance *V*, standardizing its total latent variance gives $\rho^{2}=V/(1+V)$. Section S6 in S1 Text gives the derivation and shows how genetic and environmental covariance contribute. For an ascertained calibration cohort, sampling weights or a retrospective likelihood recover the population fit. Applicability to a target GWAS depends on prevalence, covariate measurement, ancestry, age distribution, and the covariate-risk relationship. Calibration through a liability proxy also depends on its relation to the target liability.

#### Case-control sampling.

Population prevalence determines the liability threshold; the sample case fraction determines ascertainment. Under the probit generating model and sampling by case status alone, the sampled factor $\lambda_{\pi }$ averages over the ascertained covariate distribution and uses its fitted null logistic risk (Section S2 in S1 Text). A correctly specified logistic model has a sampling-invariant slope [19], while the working logistic projection of probit risk can vary with ascertainment [20]. The fitted-variance estimate applies to population samples; in a case-control sample, $\lambda_{\pi }$ also requires the population risk model.

#### Uncertainty.

With a known external factor, dividing the bias-subtracted coefficient and its standard error (SE) by the same factor preserves the Wald statistic. Estimated prevalence, explained variance, and bias slopes contribute additional variance. Shared calibration or bias estimates also induce covariance across corrected SNP effects, including for instruments independent in LD.

Joint delta-method calculations or resampling propagate this uncertainty, using a GWAS covariance appropriate for the sampling design and working logistic fit. Section S5 in S1 Text gives the sandwich covariance and resampling procedures; Algorithm A in S1 Text summarizes implementation.

### 2.5 Rescaling downstream analyses

For univariable MR, let *X* denote the exposure and *Y* the outcome. We write the population association relationship as $\beta_{G_{i}Y}=\theta \beta_{G_{i}X}^{liab}+\nu_{i}$, where $\nu_{i}$ represents departures from proportionality, such as horizontal pleiotropy. Under the valid-instrument model, $\nu_{i}=0$, and the exposure and outcome associations describe compatible causal pathways [21]. Estimated associations also contain sampling error. The inverse-variance weighted (IVW) estimator is


$$\begin{array}{c} {\hat{\theta }}_{IVW}\;=\;\frac{\sum_{i}w_{i}\;{\hat{\beta }}_{G_{i}Y}\;{\hat{\beta }}_{G_{i}X}^{liab}}{\sum_{i}w_{i}\;({\hat{\beta }}_{G_{i}X}^{liab}{)}^{2}}, \end{array}$$ (8)

where $w_{i}=1/SE({\hat{\beta }}_{G_{i}Y}{)}^{2}$ are the usual first-order inverse outcome-variance weights.

With fixed conditional-to-marginal factor ratios $\kappa_{X}$ for the exposure and $\kappa_{Y}$ for the outcome,


$$\begin{array}{c} {\hat{\theta }}_{cond}=\frac{\kappa_{X}}{\kappa_{Y}}{\hat{\theta }}_{marg}, \end{array}$$

for the same selected instruments and first-order IVW weighting. An unchanged side has factor one. Rescaling all outcome SEs by the same outcome factor multiplies every weight by a common constant, which cancels from the fit.

The same argument extends to multivariable MR (MVMR), with one factor per exposure. For fixed weights and full column rank, the coefficient vector rescales componentwise even when the exposure columns are correlated. Section S9 in S1 Text gives the matrix identity and downstream calculations.

Dividing all direct SNP effects by κ changes their genetic-component variance by $1/\kappa^{2}$. For total SNP heritability, the target also includes mediated pathways and their covariance with the direct component. For a polygenic score (PGS) constructed from the same coefficients and evaluated in the same sample, the regression slope changes inversely with the score’s scale, while ranking and *R*^2^ are preserved. Absolute-risk prediction additionally requires a calibrated intercept, outcome model, and target population.

## 3 Results

### 3.1 Conditional risk heterogeneity increases the conversion factor

We first evaluated how the conversion depends on disease prevalence and covariate-explained liability variance (Fig 2A). The ratio $\kappa_{cond}$ increases with $\rho^{2}$ at fixed prevalence and is larger for more common diseases within the evaluated range. The full grid is in Table B in S1 Text.

**Fig 2:**
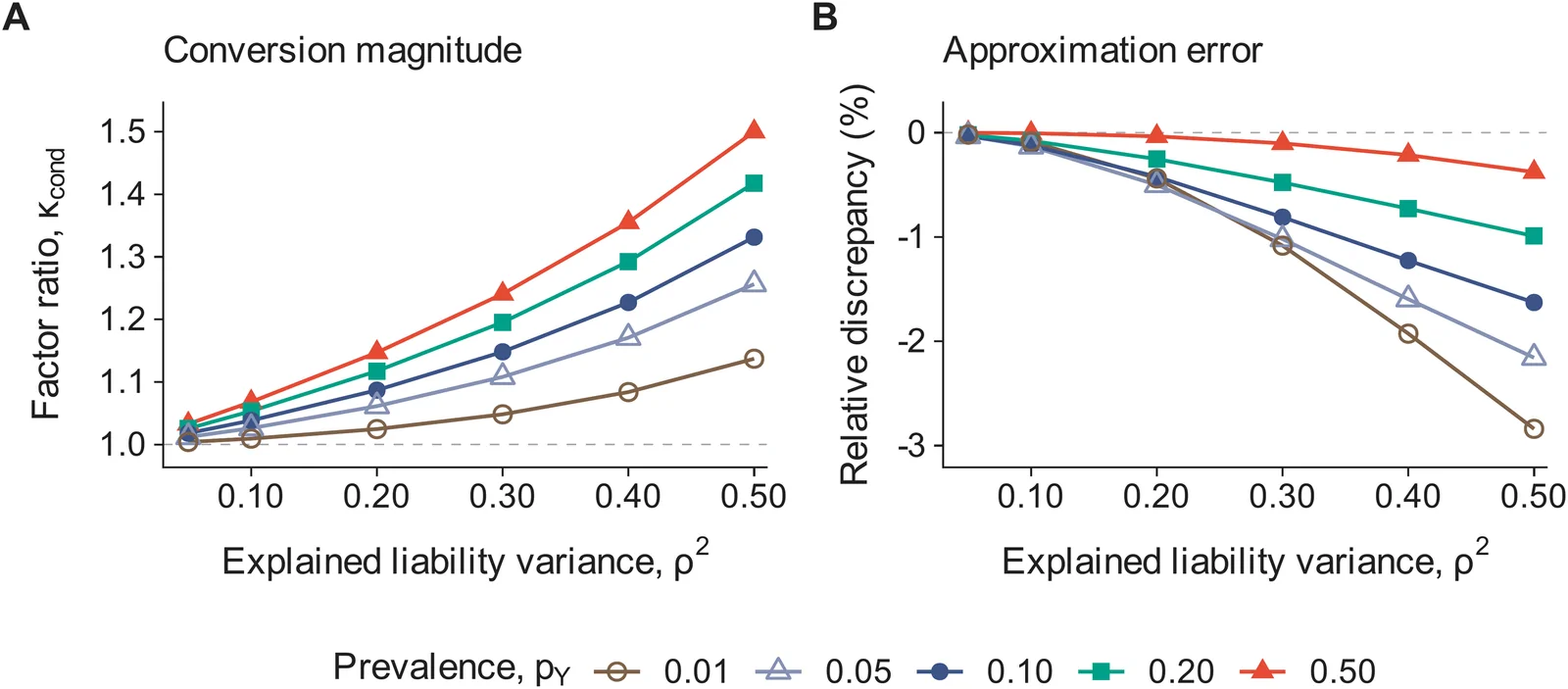
Population conversion factors. **(A)** Ratio $\lambda_{cond}/\lambda_{marg}$. **(B)** Relative discrepancy, $100(\lambda_{cond}/\lambda_{logit}-1)$. Points show the evaluated grid; lines connect them. Values: Tables B and C in S1 Text.

We next compared the approximation with the population working-logistic factor (Fig 2B). The approximation was smaller than the working-logistic factor in every setting, by less than 3%, and the discrepancy increased with explained liability variance as the two Bernoulli-variance denominators diverged. Table C in S1 Text gives the numerical comparison.

### 3.2 Conditional conversion reduces scaling bias

We evaluated conversion in individual-level simulations with and without collider bias (Fig 3). In the no-collider design, the subtraction term is zero in expectation, which isolates the effect of rescaling. Marginal scaling increasingly overstated the direct effect as explained variance rose, whereas conditional scaling kept the absolute relative bias below 2% in every setting (Fig 3A). Complete results are given in Table E in S1 Text.

**Fig 3:**
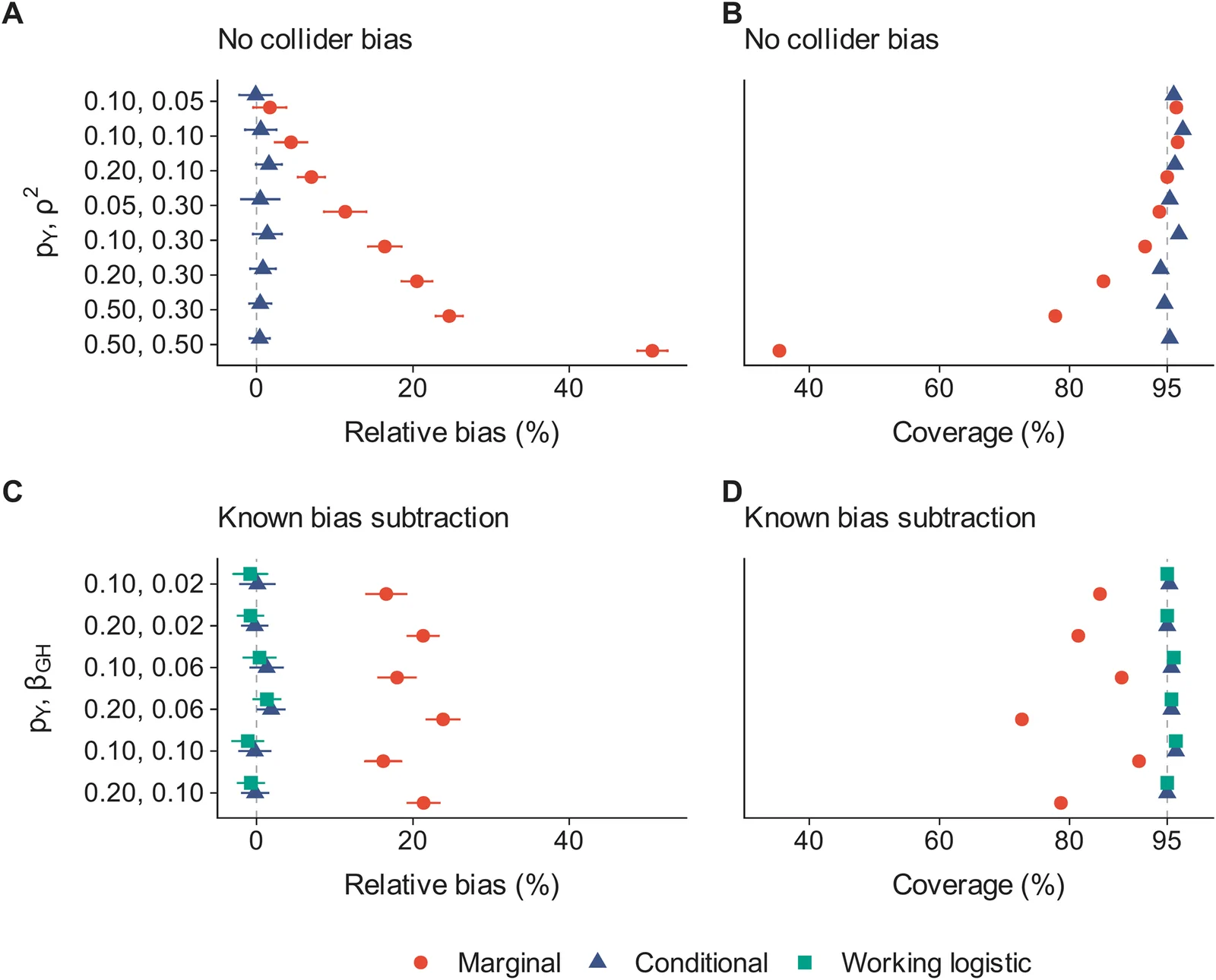
Relative bias and coverage of 95% intervals. **(A,B)** No collider term (*n* = 20,000; 500 replicates per setting). **(C,D)** Known bias subtraction (*n* = 30,000; 300 replicates per setting). Bias bars: ±1.96 Monte Carlo SE. Calibration and subtracted bias are fixed. Details: Section S4 in S1 Text.

Conditional scaling gave coverage close to the nominal level, whereas marginal-scaled coverage deteriorated as explained variance increased (Fig 3B). The small remaining conditional-scaled bias is consistent with the working-variance discrepancy in Fig 2B and Monte Carlo error. These intervals treat the conversion inputs as fixed.

In the explicit-collider design, we subtracted the known first-order log-odds bias, $\lambda_{logit}b_{lin}\beta_{GH}$, and converted the same numerator with each factor (Fig 3C and 3D). Marginal scaling left substantial bias after subtraction, whereas conditional and working-logistic scaling both gave small residual bias and coverage close to the nominal level. This design also tests the shared factor in Eq (4). The collider contribution varied across settings. A different factor for that contribution would have left a bias that changed with $\beta_{GH}$; the remaining bias showed no such trend. The complete design and Monte Carlo uncertainty are in Section S4 in S1 Text.

### 3.3 Calibration error changes the inferred liability effect

The preceding simulations used the generating calibration inputs. To examine errors in those inputs, we held the disease model fixed and varied the assumed explained variance (Fig 4). Underestimating the explained variance gives too small a factor and overstates the direct effect. Overestimating the explained variance reverses the sign of the error. The complete sensitivity grid is in Table F in S1 Text.

**Fig 4:**
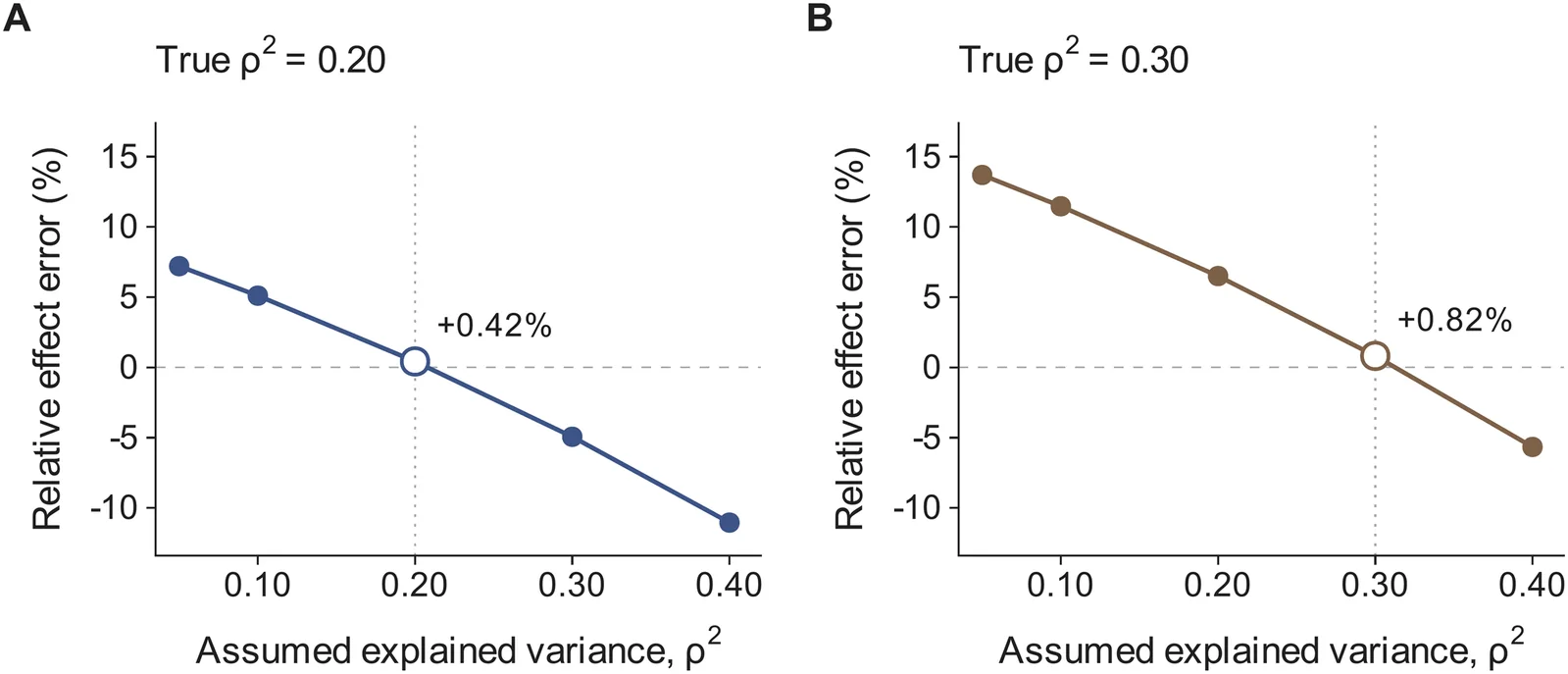
Calibration sensitivity at $p_{Y}=0.10$. Relative effect error is shown for true $\rho^{2}=0.20$ (A) and 0.30 **(B)**. Outlined points mark matched calibration, where approximation error remains. Details: Section S7 in S1 Text.

When the assumed explained variance matched its generating value, only the small working-variance approximation error remained. Errors from misspecified calibration were larger, making calibration the more consequential source of scale error in these settings.

Prevalence sensitivity and case-control calculations are in Sections S10.1 and S2 in S1 Text.

### 3.4 Rescaling reduces bias in MR simulations

We next examined univariable and multivariable MR using summary statistics generated with $\lambda_{cond}$ and matched calibration inputs (Sections S9.3 and S10.1 in S1 Text). These designs isolate downstream estimation after conversion.

In the T2D-to-coronary artery disease (CAD) experiment, with the bias slope fitted by regression, conditional scaling substantially reduced absolute IVW bias across the evaluated designs (Table J in S1 Text).

The remaining attenuation is the regression dilution expected from sampling error in the exposure associations, which the first-order IVW estimator does not model; the robust adjusted profile score estimator (MR-RAPS) accounts for it [22]. For exposure associations $\lambda \gamma_{k}+e_{k}$ divided by λ, the IVW estimate converges to $\theta \lambda^{2}\text{Var}(\gamma )/\{\lambda^{2}\text{Var}(\gamma )+\text{Var}(e)\}$. Predicted attenuation agreed with the simulations and explained the remaining undercoverage: IVW intervals treat exposure associations and the fitted bias slope as fixed (Section S10.2 in S1 Text).

The two-exposure MVMR experiment showed the same pattern. Conditional-prevalence rescaling changed each coefficient by its exposure-specific factor, as the fixed-weight identity predicts, and the remaining underestimation agreed with the predicted regression dilution (Table G in S1 Text). Tables H and I in S1 Text give the corresponding fixed-scale calculations for direct genetic-component variance and PGS regression slopes.

### 3.5 Estimated conversion factors recover the scale of direct effects

To examine the full estimation procedure, we combined bias-slope estimation and liability conversion in simulated population and case-control GWAS (Fig 5; Section S11 in S1 Text). Each replicate included an adjusted logistic GWAS with binomial genotypes under the collider model of Fig 1. Independent samples supplied the covariate and outcome associations and a calibration cohort. We estimated the bias slope using indexevent CWLS [5,7] and Slope-Hunter [6]. Conversion factors came from the fitted GWAS model or cohort calibration; the latter used the specified population prevalence. We assessed overall scale accuracy by regressing the converted effects on their generating values through the origin, and evaluated coverage using approximate marginal SEs. The MR analysis used outcome associations generated in proportion to the direct liability effects.

**Fig 5:**
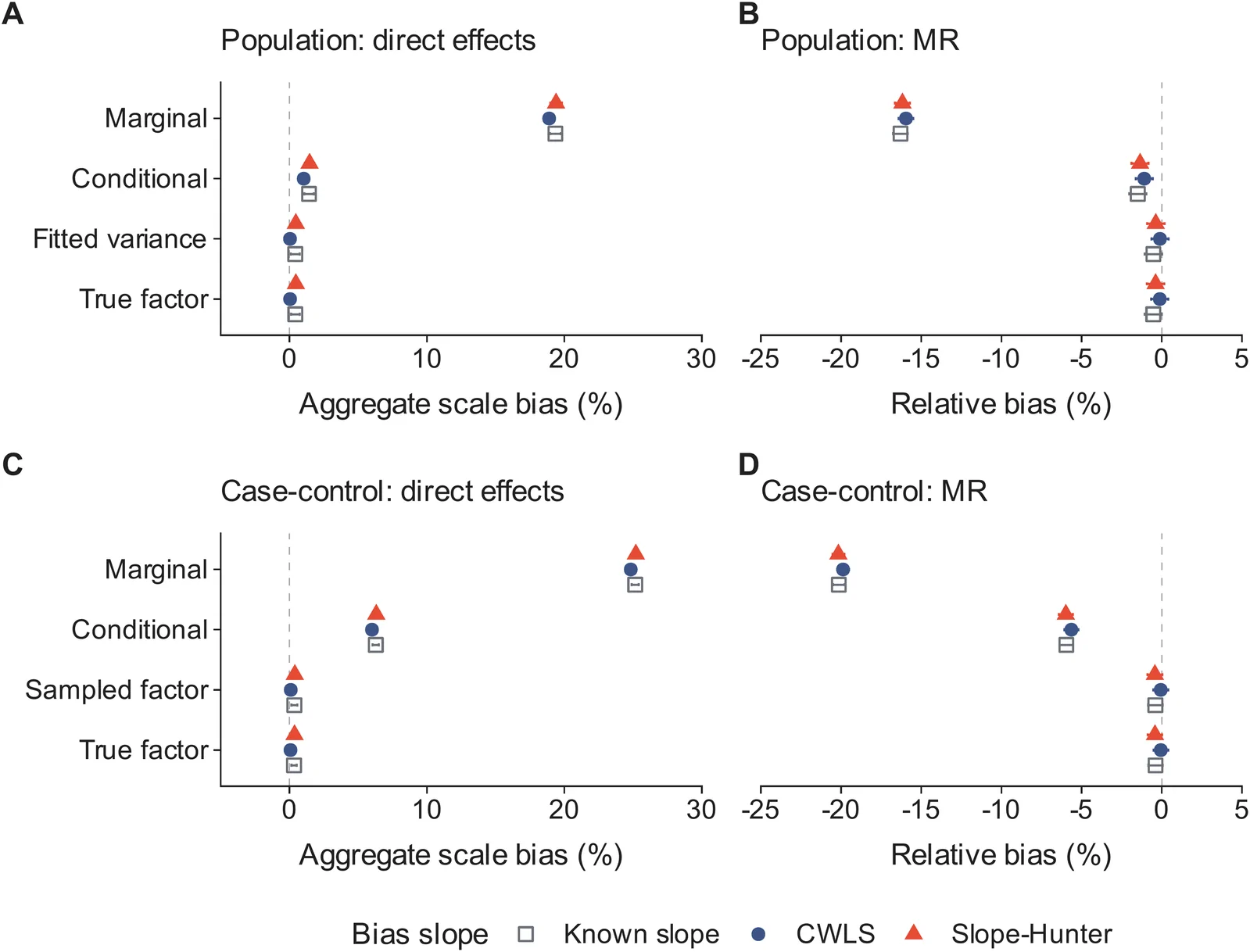
Estimated bias subtraction and liability conversion. Aggregate scale bias of direct effects (A,C) and relative bias of MR-RAPS estimates **(B,D)**, in population (A,B) and case-control (C,D) samples. Known-slope and true-factor rows are benchmarks. Bars: ±1.96 Monte Carlo SE over 200 replicates. Design and metrics: Section S11 in S1 Text.

In the population sample, marginal conversion inflated the overall scale of the direct effects. The fitted-variance factor nearly eliminated this scale bias and gave per-variant coverage close to the nominal level (Fig 5A). In the case-control sample, the sampled factor similarly recovered the scale of the direct effects, whereas the population factor retained appreciable bias (Fig 5C). With these design-specific factors, MR-RAPS estimates were also close to the generating association ratio (Fig 5B and 5D). Table M in S1 Text gives the estimates, Monte Carlo uncertainty, and coverage for each method.

CWLS recovered the log-odds bias slope closely, whereas Slope-Hunter overestimated its magnitude. Both methods recovered the overall scale of direct effects in this design, where direct effects and covariate associations were independently generated. A slope error still changes an individual corrected effect in proportion to its association with the covariate; its contribution to the aggregate scale can average toward zero across variants.

### 3.6 Sensitivity calculations using public GWAS data

At the primary assumed calibration, conditional-prevalence conversion reduced the marginal-scaled BMI-to-T2D estimate by 13%, with the same proportional change in its SE. Because the outcome GWAS conditions on BMI, this estimate retains the collider contribution and does not estimate a total BMI effect; it shows the size of the scale change for an adjusted outcome (Section S10.4.2 in S1 Text).

For Alzheimer’s disease (AD), the hypothetical Gaussian-risk inputs gave conversion factors close to the marginal factor (Table L in S1 Text). The accompanying linkage disequilibrium score regression (LDSC) estimate used the Bellenguez et al. release, which combines clinical and proxy-AD cases without *APOE* adjustment; it is reported separately in Section S10.4.1 in S1 Text.

## 4 Discussion

For small genetic effects under a Gaussian liability model, the direct effect and collider contribution share one conversion factor in adjusted binary GWAS. This connects existing collider-bias corrections to direct liability effects and explains why prevalence alone can overstate their magnitude.

The sensitivity analyses identify calibration as a larger source of scale error than the conditional-prevalence approximation over the evaluated settings. Information about the covariate-risk relationship in the target population is therefore central to interpreting adjusted effects.

Bias-slope accuracy and aggregate scale accuracy assess different parts of the correction. Slope error can distort individual associations even when its average contribution across variants is small, as in our simulations with independently generated direct and covariate effects. The choice of bias estimator remains tied to its identifying assumptions and the genetic architecture. Section S11 in S1 Text specifies the marginal SE approximation used here and its omitted covariance terms.

Rescaling changes effect units while preserving genetic correlation, PGS ranking, and predictive *R*^2^ (Tables H and I in S1 Text). The MR simulations distinguish this scale change from regression dilution: liability conversion removed scale bias, while MR-RAPS also reduced attenuation from exposure-association error [22].

Fig 1 represents a hypothetical causal structure. Its application draws on biological knowledge and study design, since genetic summary associations alone do not establish the pathways involving unmeasured confounders. We assume that the focal instrument is independent of the hidden confounder. This includes heritable confounders whose relevant variation is independent of that instrument. Discrete or strongly skewed covariates can be examined through the risk averages in Section S1.6 in S1 Text. Our case-control extension considers sampling by disease status. Matched samples, genotype-dependent selection, mixed-model GWAS, and heterogeneous meta-analyses are settings for further score derivations.

Covariate choice follows the biological question and genetic architecture and can increase or reduce association-test power [12,13]. For studies targeting direct liability effects, a useful report gives the adjusted covariates, population prevalence, sampling design, bias estimator, and conversion factor with its calibration source and uncertainty. Calibration reflects the full observational risk relationship, including genetic and environmental associations that may reinforce or offset each other. Reporting these quantities for the same population and pathways supports comparison and reuse of adjusted GWAS associations.

## Supporting information

S1 Text Supplementary methods, proofs, Tables A–M, and Algorithm A. (PDF)

S1 Data Numerical data underlying the figures, supplementary tables, and reported numerical checks. (XLSX)

S1 Checklist STROBE-MR reporting checklist. Adapted from Skrivankova et al. (2021), https://doi.org/10.1001/jama.2021.18236; source checklist: https://www.strobe-mr.org/download/strobe-mr-checklist-fillable/. (PDF)
