# Supplementary material for: Conditional-prevalence correction for liability-scale analyses of covariate-adjusted binary GWAS
Source: PLoS Genet. 2026 Oct 30;22(10):e1012348. doi: 10.1371/journal.pgen.1012348 (PMC13722303; doi:10.1371/journal.pgen.1012348)
Supplement: Supplementary file 1 [file pgen.1012348.s001.pdf]

S1 Text

Conditional-prevalence correction for liability-scale analyses of covariate-adjusted binary GWAS

Siyi Chen

School of Public Health, LSU Health Sciences Center, New Orleans

Contents

Note S0: Notation . . . 2
S1 Conditional liability-to-logit conversion . . . 3
S1.1 Collider model . . . 3
S1.2 Population logistic-regression score equation . . . 4
S1.3 Common factor for the direct and collider terms . . . 5
S1.4 Numerator invariance under Gaussian $M(\mathbf{H})$ . . . 5
S1.5 Error in the conditional-prevalence approximation . . . 6
S1.6 Non-Gaussian covariate-risk distributions . . . 6
S2 Case-control sampling and the working-logistic factor . . . 7
S3 Identification and estimation of the bias slope . . . 8
S4 Individual-level simulation designs . . . 9
S4.1 No-collider simulation . . . 9
S4.2 Explicit-collider simulation . . . 9
S5 Variance, fixed-scale inference, and resampling . . . 10
S6 Calibration from population data . . . 11
S6.1 Genetic and environmental contributions to calibration . . . 11
S6.2 Population probit calibration . . . 12
S6.3 Fitted-variance factor in population samples . . . 12
S7 Sensitivity to calibration . . . 12
S8 Relation to earlier work . . . 13
S8.1 Liability-scale heritability . . . 13
S8.2 Risk-informed association testing . . . 13
S8.3 Covariate adjustment and power . . . 13
S8.4 Liability conversion in downstream analyses . . . 14
S9 Downstream analyses: MR, genetic variance, and polygenic scores . . . 14
S9.1 Univariable Mendelian randomization . . . 14
S9.2 Multivariable MR and fixed-weight rescaling . . . 15
S9.3 Multivariable MR simulations . . . 15
S9.4 Direct genetic-component variance . . . 16
S9.5 Polygenic-score scaling . . . 17
S9.6 Genetic correlation . . . 17
S10 Disease examples and public-data analyses . . . 17
S10.1 Illustrative simulation: BMI-adjusted T2D in MR with CAD . . . 17
S10.2 Regression dilution in the MR simulations . . . 18
S10.3 Hypothetical calculation motivated by Alzheimer's disease . . . 19
S10.4 Analyses of AD and BMI-adjusted T2D GWAS . . . 20
S11 End-to-end validation . . . 21
S12 Implementation and reproducibility . . . 23

Note S0: Notation

Table A. Notation.

Symbol	Meaning
Y, Y^*	Binary disease outcome; underlying continuous liability (Y^* at unit variance)
p_Y, t_Y	Population disease prevalence; liability threshold $t_Y = \Phi^{-1}(1 - p_Y)$
G_i	Standardized genetic variant i
$\mathbf{H}, \mathbf{J}$	Adjusted covariates; additional design covariates (conditioned on here)
U	Unmeasured confounder of $\mathbf{H}$ and Y
γ_i	Liability-scale direct genetic effect of G_i on Y^* (target estimand)
$\beta^C_{G_iY}$	Covariate-adjusted log-odds GWAS coefficient
$\beta_{G_i\mathbf{H}}$	SNP-covariate effect vector (linear regression)
$M(\mathbf{H})$	Population-projected covariate liability component, $M = \boldsymbol{\delta}_H^{\text{eff}\top}\mathbf{H}$
ρ^2	Variance of $M(\mathbf{H})$ at the focal-SNP null
σ^2_η	Variance of structural noise η in the liability equation
τ	Conditional SD of liability given $\mathbf{H}$ at the focal-SNP null, $\tau = \sqrt{1-\rho^2}$
$\boldsymbol{\delta}^*_H$	Direct partial liability-scale effects of $\mathbf{H}$ given G_i and U
$\boldsymbol{\delta}^{\text{eff}}_H$	Population partial regression slope of liability on $\mathbf{H}$ given G_i
$\mathbf{b}_{\text{lin}}$	Signed additive collider slope, $-\alpha_U V_0^{-1}\boldsymbol{\alpha}_{UH}$
$b_{\text{lin}}, b_{\text{logOR}}$	Linear-scale and log-odds-scale bias slopes; $b_{\text{logOR}} = \lambda_{\text{logit}}\, b_{\text{lin}}$
λ_{marg}	Marginal liability-to-logit factor $= \phi(t_Y)/[p_Y(1-p_Y)]$
$\lambda_{\text{logit}}, \lambda_\pi$	Exact first-order population and sampled working-logistic factors
λ_{cond}	Conditional-prevalence approximation to the working-logistic factor
κ_{cond}	Ratio of the conditional-prevalence and marginal factors, $\lambda_{\text{cond}}/\lambda_{\text{marg}} \geq 1$
$\widehat{\beta}^{\text{marg}}_{G_iY^*}, \widehat{\beta}^{\text{cond}}_{G_iY^*}$	Marginal-scaled and conditional-scaled estimators, $A_i/\widehat{\lambda}_{\text{marg}}$ and $A_i/\widehat{\lambda}_{\text{cond}}$
A_i	Bias-subtracted log-odds coefficient $= \widehat{\beta}^C_{G_iY} - \widehat{\mathbf{b}}^\top_{\text{logOR}}\widehat{\boldsymbol{\beta}}_{G_i\mathbf{H}}$
ϵ_i	Size of the focal genetic effects, $(\gamma_i^2 + \lVert\boldsymbol{\beta}_{G_i\mathbf{H}}\rVert_2^2)^{1/2}$
ν_i	Population residual in the MR relationship; zero under the valid-instrument model

S1 Conditional liability-to-logit conversion

S1.1 Collider model

Suppressing the focal-variant index, the model in main-text Section 2.1 is

$$\mathbf{H} = \boldsymbol{\beta}_{G\mathbf{H}}\, G + \boldsymbol{\alpha}_{UH} U + \boldsymbol{\zeta}, \quad \boldsymbol{\zeta} \sim \mathcal{N}(\mathbf{0}, \boldsymbol{\Sigma}_\zeta), \quad U \sim \mathcal{N}(0,1), \tag{S1.1}$$

$$Y^* = \gamma G + \boldsymbol{\delta}_H^{*\top} \mathbf{H} + \alpha_U U + \eta, \quad \eta \sim \mathcal{N}(0, \sigma_\eta^2), \quad \mathrm{Var}(Y^*) = 1, \tag{S1.2}$$

$$Y = \mathbf{1}\{Y^* > t_Y\}, \quad t_Y = \Phi^{-1}(1 - p_Y).$$

Assume mutually independent Gaussian $G, U, \boldsymbol{\zeta}, \eta$, with centered, standardized G, U and positive definite $\boldsymbol{\Sigma}_\zeta$. Background genetic contributions to $\boldsymbol{\zeta}$ must also be independent of the focal marker and U.

Joint normality gives

$$\mathbb{E}[U \mid G, \mathbf{H}] \;=\; \mathbf{c}^\top (\mathbf{H} - \boldsymbol{\beta}_{G\mathbf{H}}\, G),$$

where $\mathbf{c} = \mathrm{Var}(\boldsymbol{\alpha}_{UH} U + \boldsymbol{\zeta})^{-1} \boldsymbol{\alpha}_{UH}$. Substitution gives

$$\mathbb{E}[Y^* \mid G, \mathbf{H}] \;=\; (\gamma + \mathbf{b}_{\mathrm{lin}}^\top \boldsymbol{\beta}_{G\mathbf{H}})\, G + \boldsymbol{\delta}_H^{\mathrm{eff}\top} \mathbf{H},$$

where $\mathbf{b}_{\mathrm{lin}} = -\alpha_U \mathbf{c}$ is the signed additive liability-scale bias slope and $\boldsymbol{\delta}_H^{\mathrm{eff}} = \boldsymbol{\delta}_H^* + \alpha_U \mathbf{c}$ is the population partial regression slope of Y^* on $\mathbf{H}$ given G. The conditional variance of Y^* given $(G, \mathbf{H})$ is $\alpha_U^2 (1 - \boldsymbol{\alpha}_{UH}^\top \mathbf{c}) + \sigma_\eta^2$.

At $(\gamma, \boldsymbol{\beta}_{G\mathbf{H}}) = (0, \mathbf{0})$, define the covariate predictor

$$M(\mathbf{H}) \;=\; \boldsymbol{\delta}_H^{\mathrm{eff}\top} \mathbf{H}, \quad \rho^2 = \mathrm{Var}\{M(\mathbf{H})\} = \boldsymbol{\delta}_H^{\mathrm{eff}\top} \boldsymbol{\Sigma}_{\mathbf{H}} \boldsymbol{\delta}_H^{\mathrm{eff}}.$$

Joint normality and the law of total variance give

$$\mathrm{Var}(Y^* \mid \mathbf{H}) \;=\; 1 - \rho^2 \;\equiv\; \tau^2$$

and the conditional disease risk is

$$p(\mathbf{H}) \;=\; \Pr(Y = 1 \mid \mathbf{H}) \;=\; \Phi\!\left(\frac{M(\mathbf{H}) - t_Y}{\tau}\right),$$

The conditional SD τ includes residual confounder variation as well as structural noise: $\sigma_\eta^2 = \tau^2 - \alpha_U^2 (1 - \boldsymbol{\alpha}_{UH}^\top \mathbf{c})$ at the null.

For the scalar collider simulation, unit total liability variance requires

$$\begin{aligned} 1 = \mathrm{Var}(Y^*) &= (\gamma + \delta_H^* \beta_{GH})^2 + (\delta_H^* \alpha_{UH} + \alpha_U)^2 \\ &\quad + (\delta_H^*)^2 \sigma_\zeta^2 + \sigma_\eta^2. \end{aligned}$$

We set σ_η^2 from this expression. With the other structural coefficients fixed, its change from the null is second order in the focal effects.

S1.2 Population logistic-regression score equation

We expand the adjusted working-logistic coefficient jointly in γ and $\boldsymbol{\beta} = \boldsymbol{\beta}_{G\mathbf{H}}$. Write $H = \mathbf{H}$, $V_0 = \boldsymbol{\alpha}_{UH}\boldsymbol{\alpha}_{UH}^\top + \boldsymbol{\Sigma}_\zeta$, and $a = \gamma + \mathbf{b}_{\text{lin}}^\top \boldsymbol{\beta}$.

Let $\epsilon = (\gamma^2 + \|\boldsymbol{\beta}\|_2^2)^{1/2}$. Assume $0 < p_Y < 1$, $0 \le \rho^2 < 1$, positive structural noise variance at the null, and a finite null logistic fit with nonsingular information. We fix the non-focal coefficients and adjust σ_η^2 as above to maintain unit liability variance; the conditional variance remains positive locally. Expressing H through fixed independent Gaussian variables bounds the first two score derivatives by integrable Gaussian moments. The expected score is therefore twice continuously differentiable, with coefficient Jacobian equal to minus the logistic information matrix. The implicit function theorem gives a twice continuously differentiable fitted coefficient and a second-order remainder. The conditional risk expands as

$$\Pr(Y = 1 \mid G, H) = p(H) + aG\dot{p}(H) + O(\epsilon^2)$$

in the integrated score equations, with $p(H) = \Phi\{(M(H) - t_Y)/\tau\}$ and $\dot{p}(H) = \tau^{-1}\phi\{(M(H) - t_Y)/\tau\}$, the derivative with respect to a liability shift. For parameter vector $\boldsymbol{\vartheta} = (\alpha, \beta_G, \boldsymbol{d}^\top)^\top$, put $\boldsymbol{X} = (1, G, H^\top)^\top$ and $q_{\boldsymbol{\vartheta}}(G, H) = \operatorname{expit}(\alpha + \beta_G G + \boldsymbol{d}^\top H)$. The score equations are

$$\begin{aligned}
\mathbb{E}[Y - q_{\boldsymbol{\vartheta}}(G, H)] &= 0, \\
\mathbb{E}[G\{Y - q_{\boldsymbol{\vartheta}}(G, H)\}] &= 0, \\
\mathbb{E}[H\{Y - q_{\boldsymbol{\vartheta}}(G, H)\}] &= \mathbf{0}.
\end{aligned}$$

At the null, G is independent of (Y, H) and has mean zero and variance one, so the null SNP coefficient is zero. Write the null fit as $q_0(H) = \operatorname{expit}(\alpha_0 + \boldsymbol{d}_0^\top H)$ and $v_0(H) = q_0(H)\{1 - q_0(H)\}$. Then

$$q_{\boldsymbol{\vartheta}}(G, H) = q_0(H) + v_0(H)(\Delta\alpha + \Delta\beta_G G + \Delta\boldsymbol{d}^\top H) + O(\epsilon^2).$$

At the null the cross-information blocks involving the SNP are zero:

$$\mathbb{E}[Gv_0(H)] = 0, \qquad \mathbb{E}[GH^\top v_0(H)] = \mathbf{0}^\top.$$

The intercept and covariate perturbations therefore cancel from the first-order SNP score.

Changing $\boldsymbol{\beta}$ also changes the joint distribution of (G, H). Joint normality and $\operatorname{Var}(H) = V_0 + \boldsymbol{\beta}\boldsymbol{\beta}^\top$ give

$$\mathbb{E}[G \mid H] = \boldsymbol{\beta}^\top V_0^{-1} H + O(\|\boldsymbol{\beta}\|_2^3)$$

in the corresponding integrated moments. Its first-order contribution to the SNP score is

$$\mathbb{E}[G\{p(H) - q_0(H)\}] = \boldsymbol{\beta}^\top V_0^{-1}\mathbb{E}[H\{p(H) - q_0(H)\}] + O(\epsilon^2) = O(\epsilon^2),$$

because the null covariate score is zero. The first-order SNP score is therefore

$$0 = a\mathbb{E}[\dot{p}(H)] - \Delta\beta_G \mathbb{E}[v_0(H)] + O(\epsilon^2).$$

Hence

$$\beta_G = \frac{\mathbb{E}[\dot{p}(H)]}{\mathbb{E}[q_0(H)\{1 - q_0(H)\}]}(\gamma + \boldsymbol{b}_{\text{lin}}^\top \boldsymbol{\beta}) + O(\epsilon^2). \tag{S1.3}$$

The Gaussian model also reduces the null logistic fit to a one-dimensional calculation. For $\rho^2 > 0$,

$$\mathbb{E}[H \mid M] = V_0 \boldsymbol{\delta}_H^{\text{eff}} M/\rho^2.$$

Choose $q_0(M) = \text{expit}(\alpha_0 + d_M M)$ to satisfy $\mathbb{E}[p - q_0] = \mathbb{E}[M(p - q_0)] = 0$. The conditional expectation above then gives $\mathbb{E}[H(p - q_0)] = \mathbf{0}$. Thus the one-dimensional fit solves the full covariate score, and strict convexity identifies the same null logistic projection. When $\rho^2 = 0$, risk is constant and the fit contains only an intercept. Under case-control sampling, the same argument applies to the population-weighted residual $rp - sq_\pi$ in Section S2.

S1.3 Common factor for the direct and collider terms

Both γ and $\mathbf{b}_{\text{lin}}^\top \boldsymbol{\beta}$ shift risk through $G\dot{p}(H)$, so Eq (S1.3) assigns them the same factor.

S1.4 Numerator invariance under Gaussian $M(\mathbf{H})$

Lemma S1. *Suppose $M \sim \mathcal{N}(0, \rho^2)$ with $\rho^2 \in [0, 1)$, and let $\tau = \sqrt{1 - \rho^2}$. Then*

$$\mathbb{E}_M\big[\tau^{-1}\, \phi((M - t_Y)/\tau)\big] \;=\; \phi(t_Y) \quad \textit{for all } \rho^2 \in [0, 1).$$

Proof. Let $Z \sim \mathcal{N}(0, \tau^2)$ be independent of M. Since $M + Z \sim \mathcal{N}(0, 1)$, convolution gives

$$\mathbb{E}_M[\tau^{-1}\phi\{(M - t_Y)/\tau\}] = \int f_M(m) f_Z(t_Y - m)\, \mathrm{d}m = f_{M+Z}(t_Y) = \phi(t_Y)$$

for $\rho > 0$. At $\rho = 0$, $M = 0$ and $\tau = 1$, so the identity holds directly. □

Applying Lemma S1 to the numerator of Eq (S1.3) gives

$$\lambda_{\text{logit}} = \frac{\phi(t_Y)}{\mathbb{E}[q_0(1 - q_0)]}, \qquad \lambda_{\text{cond}} = \frac{\phi(t_Y)}{\mathbb{E}[p(1 - p)]}.$$

Concavity of $u(1 - u)$ gives

$$\mathbb{E}_{\mathbf{H}}\{p(\mathbf{H})[1 - p(\mathbf{H})]\} \;\le\; \mathbb{E}_{\mathbf{H}}[p(\mathbf{H})]\big\{1 - \mathbb{E}_{\mathbf{H}}[p(\mathbf{H})]\big\} \;=\; p_Y(1 - p_Y),$$

with strict inequality whenever $\text{Var}_{\mathbf{H}}[p(\mathbf{H})] > 0$. Thus $\kappa_{\text{cond}} \ge 1$, with equality only when $\rho^2 = 0$.

S1.5 Error in the conditional-prevalence approximation

Table B gives the factor grid for main-text Fig 2A.

Table B. Conditional-prevalence correction factor $\kappa_{\text{cond}} = \lambda_{\text{cond}}/\lambda_{\text{marg}}$ across disease prevalence p_Y and covariate-explained liability variance ρ^2. Computed by 200-point Gauss-Hermite quadrature of main-text Eq (6).

ρ^2	$p_Y = 0.50$	$p_Y = 0.20$	$p_Y = 0.10$	$p_Y = 0.05$	$p_Y = 0.01$
0.05	1.033	1.026	1.018	1.012	1.004
0.10	1.068	1.053	1.038	1.026	1.009
0.20	1.147	1.117	1.087	1.061	1.025
0.30	1.241	1.195	1.148	1.108	1.048
0.40	1.355	1.292	1.227	1.171	1.084
0.50	1.500	1.418	1.331	1.256	1.137

Replacing the fitted logistic variance $q_0(1-q_0)$ by the true risk variance $p(1-p)$ gives relative discrepancy

$$\frac{\lambda_{\text{cond}} - \lambda_{\text{logit}}}{\lambda_{\text{logit}}} = \frac{\mathbb{E}[q_0(1-q_0)]}{\mathbb{E}[p(1-p)]} - 1.$$

We solved the null logistic score equations to compare these averages (Table C).

Table C. Probit-logit gap: relative discrepancy between the conditional-prevalence factor λ_{cond} and the working-logistic factor λ_{logit} computed by deterministic Gauss-Hermite quadrature of the population score equations. Values are $|\lambda_{\text{cond}} - \lambda_{\text{logit}}|/\lambda_{\text{logit}} \times 100\%$.

ρ^2	$p_Y = 0.50$	$p_Y = 0.20$	$p_Y = 0.10$	$p_Y = 0.05$
0.05	0.00	0.02	0.03	0.03
0.10	0.01	0.08	0.12	0.13
0.20	0.03	0.25	0.42	0.50
0.30	0.10	0.48	0.81	1.02
0.40	0.21	0.73	1.23	1.60
0.50	0.38	0.99	1.63	2.16

This population-level discrepancy increased with ρ^2. In the primary T2D design, λ_{cond} was about 0.8% below λ_{logit}.

S1.6 Non-Gaussian covariate-risk distributions

If M is not Gaussian, calibrate t to satisfy

$$\mathbb{E}_M\left[\Phi\left(\frac{M-t}{\tau}\right)\right] = p_Y.$$

For support points m_j with masses ω_j, expectations become sums with weights ω_j. The conditional-prevalence approximation is then

$$\lambda_{\text{cond}}^{\text{disc}} = \frac{\sum_j \omega_j \tau^{-1}\phi\{(m_j - t)/\tau\}}{\sum_j \omega_j p_j(1-p_j)}, \qquad p_j = \Phi\{(m_j - t)/\tau\}.$$

For non-Gaussian M, the numerator depends on the full calibrated risk distribution and the working-logistic denominator on the fitted covariate coding. These averages convert an independent small focal shift. Collider composition additionally requires the conditional expectation and joint-distribution expansion.

Mixed continuous-discrete covariates. For mixed covariates, average the numerator and denominator over their joint distribution, using conditional integration within strata. Fit the logistic nuisance parameters in the pooled or stratified model used for the GWAS.

S2 Case-control sampling and the working-logistic factor

Let $0 < \pi < 1$ be the fraction of cases in the sample. Suppose individuals are sampled with probabilities proportional to r for cases and one for controls, where $r = \pi(1 - p_Y)/\{p_Y(1 - \pi)\}$. At the null write $p = p(H)$, $s = 1 + (r - 1)p$, and $Z = \mathbb{E}[s]$. The sampled covariate density is $f_\pi(H) = s(H)f(H)/Z$ and sampled risk is $p_\pi(H) = rp(H)/s(H)$. Let $q_\pi(H) = \operatorname{expit}(\alpha_\pi + \boldsymbol{d}_\pi^\top H)$ solve

$$\mathbb{E}[(rp - sq_\pi)(1, H^\top)^\top] = \mathbf{0}. \tag{S2.1}$$

For a small liability perturbation aG, the unnormalized sampled score numerator is

$$\mathbb{E}\left[G\left\{rp_a(G, H) - [1 + (r - 1)p_a(G, H)]q(G, H)\right\}\right],$$

where $p_a(G, H) = p(H) + aG\dot{p}(H) + O(\epsilon^2)$. Differentiation at the null produces the signal term

$$a\mathbb{E}[\dot{p}(H)\{r - (r - 1)q_\pi(H)\}],$$

while the coefficient perturbation contributes $-\Delta\beta_G\mathbb{E}[sq_\pi(1 - q_\pi)]$. The normalizing constant Z cancels. Intercept and covariate cross-blocks vanish because G is independent of the null sampled risk variables. The changing (G, H) distribution contributes, to first order, $\boldsymbol{\beta}^\top V_0^{-1}\mathbb{E}[H(rp - sq_\pi)]$, which is zero by Eq (S2.1). Therefore

$$\beta_G = \lambda_\pi(\gamma + \boldsymbol{b}_{\text{lin}}^\top\boldsymbol{\beta}) + O(\epsilon^2), \quad \lambda_\pi = \frac{\mathbb{E}[\dot{p}\{r - (r - 1)q_\pi\}]}{\mathbb{E}[sq_\pi(1 - q_\pi)]}. \tag{S2.2}$$

At $r = 1$, Eq (S2.2) reduces to Eq (S1.3). If $\rho^2 = 0$, the numerator and denominator yield λ_{marg} for any π. Otherwise the factor generally depends on π. Prentice and Pyke [1] established slope invariance for a correctly specified logistic model. Here, the logistic projection of the generating probit risk can change under outcome-dependent sampling [2]. For sampling by case status alone, we solve the intercept and slope scores at $M = \sqrt{\rho^2}z_k$ using Gaussian quadrature weights proportional to $s(M)$ and Newton updates with a line search. All checked score residuals are below 10^{-9}.

Table D. Covariate-adjusted log-OR slope in population and case-control samples. MCSE: Monte Carlo standard error. True $\gamma = 0.05$, $p_Y = 0.10$, $\rho^2 = 0.30$, $n = 20{,}000$, $R = 200$ per case fraction. The prediction uses the independently computed first-order sampled factor.

π	Mean slope	MCSE	$0.05\lambda_\pi$	λ_π
0.05	0.1119	0.0024	0.1118	2.2366
0.10	0.1118	0.0018	0.1129	2.2573
0.30	0.1168	0.0012	0.1157	2.3131
0.50	0.1188	0.0012	0.1179	2.3586

The reported GWAS SE enters the converted variance. Applying this derivation to SAIGE [3], REGENIE [4], Firth correction [5], matched sampling, or heterogeneous meta-analysis entails using their scores and sampling assumptions.

S3 Identification and estimation of the bias slope

Bias subtraction requires a consistent estimate of $\mathbf{b}_{\mathrm{logOR}}$ from the across-variant relationship

$$\beta^{C,*}_{G_i Y} = \mathbf{b}^{\top}_{\mathrm{logOR}} \boldsymbol{\beta}_{G_i \mathbf{H}} + c_i, \qquad \text{(S3.1)}$$

where $\mathbf{b}_{\mathrm{logOR}} = \lambda_{\mathrm{logit}} \mathbf{b}_{\mathrm{lin}}$ in a population sample and c_i contains the direct effect and higher-order terms. Under case-control sampling, λ_π replaces λ_{logit}. The slope is therefore estimated in the units and sampling design of the adjusted GWAS. Ordinary regression estimates this slope under predictor-residual orthogonality and appropriate treatment of measurement error. Clustering and robust methods identify the slope through alternative assumptions about the variant associations.

In a summary-statistic model generated with λ_{cond} ($K = 200$, $n = 50{,}000$, $R = 200$), three simple regressions (ordinary, residual-trimmed, and precision-weighted) underestimated the slope by 0.9–1.7% (MCSE about 0.5%), close to the 0.8% attenuation from predictor error.

Bias-slope estimator selection. The Dudbridge index-event procedure [6] estimates bias in disease progression conditional on incidence. The CWLS estimator of Cai et al. [7] accounts for measurement error in the SNP-covariate associations; its indexevent implementation is used in Section S11. Slope-Hunter [8] identifies a univariable index-event bias slope through clusters of genetic associations. The MVMR-cML procedure of Wang et al. [9] addresses multiple adjusted covariates under a continuous-outcome model. For Algorithm A, the chosen estimator must consistently identify the bias slope in adjusted log-odds units under the target GWAS's structural, dimensional, and sampling assumptions. The binary-outcome score expansion in Section S1.2 gives the composition once this slope is identified.

S4 Individual-level simulation designs

The no-collider design tests conversion alone; the known-collider design tests the common factor for direct and collider effects. Section S11 estimates both the bias slope and calibration from data.

S4.1 No-collider simulation

We simulated independent $G \sim \mathcal{N}(0,1)$ and $H \sim \mathcal{N}(0,\rho^2)$ with direct effect $\gamma = 0.05$ and residual variance $1-\rho^2-\gamma^2$. Each setting used 500 replicates of $n =$ 20,000 individuals. We fitted logistic regression of Y on G, H and divided the coefficient and model-based SE by each fixed factor. Table E reports bias as a fraction of the true effect and coverage conditional on known calibration; main-text Fig 3A and 3B uses percentages.

Table E. Per-SNP relative bias and coverage of the marginal-scaled and conditional-scaled estimators under the no-collider data-generating model. True effect $\gamma = 0.05$; $n =$ 20,000, $R = 500$ replicates per cell. Monte Carlo standard errors for relative bias in parentheses; both are fractions of the true effect. Coverage uses nominal 95% intervals and fixed nuisance parameters.

p_Y	ρ^2	Marginal bias	Conditional bias	Cov. M (%)	Cov. C (%)
0.10	0.05	+0.017 (0.011)	-0.001 (0.011)	96.4	96.0
0.10	0.10	+0.044 (0.011)	+0.005 (0.010)	96.6	97.4
0.20	0.10	+0.070 (0.009)	+0.016 (0.008)	95.0	96.2
0.05	0.30	+0.113 (0.014)	+0.005 (0.012)	93.8	95.4
0.10	0.30	+0.164 (0.011)	+0.014 (0.010)	91.6	96.8
0.20	0.30	+0.205 (0.010)	+0.008 (0.008)	85.2	94.0
0.50	0.30	+0.246 (0.009)	+0.005 (0.007)	77.8	94.6
0.50	0.50	+0.506 (0.010)	+0.004 (0.007)	35.4	95.4

S4.2 Explicit-collider simulation

We simulated the collider model in main-text Eqs (2) and (3), with $\alpha_U = 0.5$, $\alpha_{UH} = 0.4$, $\mathrm{Var}(\zeta) = 0.45$, and $\delta_H^* = 0.4$. The signed liability-scale bias slope is $b_{\mathrm{lin}} = -0.5 \cdot 0.4/0.61 \approx -0.328$, and the null covariate-explained variance is $\rho_0^2 \approx 0.323$. We set $\gamma = 0.05$, $\beta_{GH} \in \{0.02, 0.06, 0.10\}$, $p_Y \in \{0.10, 0.20\}$, $n =$ 30,000, and $R = 300$ per cell. We set the structural noise variance using Section S1.1 to give unit total liability variance.

We subtracted the known bias $\lambda_{\mathrm{logit}} b_{\mathrm{lin}} \beta_{GH}$ and divided the same numerator by each factor. Conditional-prevalence and working-logistic conversion both reduced the bias left by marginal scaling (Fig 3).

The reported SEs condition on the known bias term and conversion factors.

S5 Variance, fixed-scale inference, and resampling

Wald tests under fixed conversion factors. For a fixed conversion factor, the estimate and its SE change proportionally:

$$z = \frac{\widehat{\beta}^{\text{cond}}_{G_iY^*}}{\text{SE}(\widehat{\beta}^{\text{cond}}_{G_iY^*})} = \frac{A_i/\widehat{\lambda}_{\text{cond}}}{\text{SE}(A_i)/\widehat{\lambda}_{\text{cond}}} = \frac{A_i}{\text{SE}(A_i)},$$

which is also the marginal-scaled Wald statistic. Fixed rescaling therefore preserves significance, instrument selection, and power for the same bias-subtracted estimate and SE.

At a null with $A_i^* = 0$, the derivative of $A_i^*/\lambda_{\text{cond}}$ with respect to the factor is zero. Consistent calibration therefore does not enter the leading asymptotic variance. For independent calibration and $A_i = O_p(n^{-1/2})$, the relative plug-in variance contribution is of order $\text{Var}(\widehat{\lambda}_{\text{cond}})/\lambda^2_{\text{cond}}$. Shared data also require the covariance term below. Bias subtraction itself can change significance and instrument selection.

Covariance of the working logistic fit. For independent observations, let $X = (1, G, H^\top)^\top$ and let q be the fitted population logistic risk in the sampling distribution. A covariance estimate valid under risk-model misspecification uses

$$I = \mathbb{E}[q(1-q)XX^\top], \qquad J = \mathbb{E}[(Y-q)^2XX^\top], \qquad \text{Avar}(\widehat{\boldsymbol{\vartheta}}) = n^{-1}I^{-1}JI^{-1}.$$

Sample analogues give the sandwich covariance; the model-based form $n^{-1}I^{-1}$ requires information equality. Our simulations used model-based logistic SEs and assessed coverage. Correlated observations require a cluster-adjusted covariance.

Delta-method variance. A first-order delta-method variance for the conditional-scaled estimator is

$$\text{Var}(\widehat{\beta}^{\text{cond}}_{G_iY^*}) \;\approx\; \frac{\text{Var}(A_i)}{\widehat{\lambda}^2_{\text{cond}}} + \frac{A_i^2}{\widehat{\lambda}^4_{\text{cond}}}\,\text{Var}(\widehat{\lambda}_{\text{cond}}) - \frac{2A_i}{\widehat{\lambda}^3_{\text{cond}}}\,\text{Cov}(A_i, \widehat{\lambda}_{\text{cond}}), \tag{S5.1}$$

where $\text{Var}(A_i)$ includes uncertainty in the GWAS coefficient, SNP-covariate associations, and bias slope, together with their covariances, including those from sample overlap. For $x_i = (\beta^C_{G_iY}, \boldsymbol{b}^\top_{\text{logOR}}, \boldsymbol{\beta}^\top_{G_i\boldsymbol{H}})^\top$ with joint sampling covariance Σ_i, the subtraction gradient is

$$g_i = (1, -\boldsymbol{\beta}^\top_{G_i\boldsymbol{H}}, -\boldsymbol{b}^\top_{\text{logOR}})^\top, \qquad \text{Var}(A_i) \approx g_i^\top \Sigma_i g_i.$$

Evaluate the gradient at consistent estimates. Section S11 specifies the marginal approximation used in the simulations and the covariance terms omitted there.

If prevalence is fixed and only ρ^2 is estimated, its contribution is

$$\text{Var}(\widehat{\lambda}_{\text{cond}}) \approx \left(\frac{\partial \lambda_{\text{cond}}}{\partial \rho^2}\right)^2_{\widehat{\rho}^2} \text{Var}(\widehat{\rho}^2),$$

The derivative can be evaluated numerically, with $\mathrm{Var}(\widehat{\rho}^2)$ estimated from calibration. When prevalence or sampling parameters are estimated, include their derivatives and covariances. A shared estimated factor also induces cross-SNP covariance.

Extreme-tail calibration. We assessed 95% interval coverage. Type-I error at 5×10^{-8} requires a dedicated tail calculation or simulations with sufficient resolution.

Resampling. For aggregate analyses, resample ancestry-appropriate LD blocks, retaining all SNPs within each block, and refit the bias slope and aggregate estimator. The Berisa-Pickrell partition [10] defines European-ancestry blocks. Resample the calibration cohort separately, or account for shared participants through joint resampling or covariance estimation. For B valid replicates, estimate variance as $(B-1)^{-1}\sum_b(\widehat{\theta}^{(B)} - \overline{\theta})^2$ and increase B until Monte Carlo error is acceptable.

For a focal SNP, use the GWAS covariance or participant-level resampling; LD-block resampling targets aggregate statistics.

S6 Calibration from population data

Population prevalence sets the liability threshold, and the observed covariate-risk relationship determines ρ^2.

Multivariable extension. For correlated Gaussian covariates, ρ^2 is the variance of their population partial-regression predictor (Section S1.2). In a no-collider simulation with one, three, or five covariates ($R = 300$, $n = 20{,}000$), the absolute bias after conditional-prevalence conversion was below 0.7%.

S6.1 Genetic and environmental contributions to calibration

For unit-variance observed H, suppose $H = H_g + H_e$ and $Y^* = Y_g^* + Y_e^*$, with orthogonal genetic and environmental components. Then $\mathrm{Cov}(Y^*, H) = r_g\sqrt{h_Y^2 h_H^2} + C_e$. At the focal-SNP null the variance of the linear prediction on H is

$$\rho^2 = \left(r_g\sqrt{h_Y^2 h_H^2} + C_e\right)^2. \tag{S6.1}$$

For example, take $h_Y^2 = h_H^2 = 0.5$, $r_g = 0.5$, and $C_e = -0.25$. Both covariance matrices are positive definite, yet the genetic and environmental covariances cancel: $\rho^2 = 0$ while $r_g^2 h_Y^2 = 0.125 > 0$. If environmental covariance is zero, Eq (S6.1) equals $r_g^2 h_Y^2 h_H^2$. Calibration to the observed covariate therefore depends on both its genetic and environmental relationship with liability.

For multiple observed covariates, the corresponding linear prediction variance is $\boldsymbol{c}_Y^\top \Sigma_H^{-1} \boldsymbol{c}_Y$, where $\boldsymbol{c}_Y = \mathrm{Cov}(H, Y^*)$ is the full observational covariance vector. Its environmental component requires observational information beyond the genetic summaries.

S6.2 Population probit calibration

In a representative cohort, fit the population probit model $\Pr(Y = 1 \mid \boldsymbol{H}) = \Phi(a + \boldsymbol{u}^\top \boldsymbol{H})$, whose residual latent variance is fixed at one. If $V = \boldsymbol{u}^\top \boldsymbol{\Sigma}_H \boldsymbol{u}$, standardizing total liability gives

$$\rho^2 = \frac{V}{1+V}, \qquad \boldsymbol{\delta}_H^{\mathrm{eff}} = \frac{\boldsymbol{u}}{\sqrt{1+V}}.$$

For case-control data, use sampling weights or a retrospective likelihood. Applying the estimate to another population depends on comparable prevalence, covariate measurement, ancestry, age, and risk relationships. A liability proxy must be calibrated to the target liability.

Calibration inputs. The grids and fixed-input analyses use specified ρ^2, with $\rho^2 = 0.30$ for the primary T2D design. Section S11 estimates calibration from data.

For centered Gaussian H, the implied prevalence is $\Phi\{a/\sqrt{1+V}\}$. We can compare this with population prevalence or constrain it during fitting. Calibration should also account for measurement error in H and differences from the GWAS population.

S6.3 Fitted-variance factor in population samples

In a random population sample, Lemma S1 supplies the numerator $\phi(t_Y)$ and the fitted null GWAS risks estimate the denominator $\mathbb{E}[q_0(1-q_0)]$. Thus

$$\widehat{\lambda}_{\mathrm{logit}} = \frac{\phi(\widehat{t}_Y)}{n^{-1}\sum_{j=1}^n \widehat{q}_j(1-\widehat{q}_j)}, \qquad \widehat{t}_Y = \Phi^{-1}(1-\widehat{p}_Y).$$

This estimate uses all disease-predictive GWAS covariates, including design covariates, without separately estimating ρ^2.

We checked this estimate in the no-collider model with $n = 20{,}000$ and 50 replicates at $(p_Y, \rho^2) = (0.10, 0.30)$, $(0.05, 0.30)$, $(0.20, 0.10)$, and $(0.50, 0.50)$. Each replicate fitted logistic regression of Y on G and H and used the sample case fraction for $\widehat{p}_Y$. The mean estimate differed from λ_{logit} by 0.07–0.31%, with Monte Carlo SEs of 0.06–0.13%. In a case-control sample, the numerator of λ_π in Eq (S2.2) depends on the population risk model, so the fitted variance alone does not determine the sampled factor.

S7 Sensitivity to calibration

After first-order bias subtraction, conversion with $\lambda_{\mathrm{cond}}(p_Y, \widehat{\rho}^2)$ gives direct-effect ratio $\lambda_{\mathrm{logit}}(p_Y, \rho^2)/\lambda_{\mathrm{cond}}(p_Y, \widehat{\rho}^2)$. Table F varies the assumed calibration while holding the

generating model fixed. A scale error shifts estimates and intervals relative to the target, with greater consequences in more precise GWAS.

Table F. First-order sensitivity to the assumed ρ^2 at $p_Y = 0.10$. The generating model is fixed within each true-ρ^2 group. Entries are deterministic calculations using the working-logistic numerator factor and the assumed conditional-prevalence conversion.

True ρ^2	Assumed ρ^2	Relative bias (%)
0.20	0.05	+7.21
0.20	0.10	+5.11
0.20	0.20	+0.42
0.20	0.30	-4.94
0.20	0.40	-11.05
0.30	0.05	+13.70
0.30	0.10	+11.47
0.30	0.20	+6.50
0.30	0.30	+0.82
0.30	0.40	-5.66

The small discrepancy at matching ρ^2 reflects the working-variance approximation. At $\rho^2 = 0.30$, increasing p_Y from 0.05 to 0.15 increased κ_{cond} from 1.108 to 1.175. Table D gives the sampled factors. The T2D/CAD simulation (Section S10.1) varies the generating ρ^2 together with its calibration input.

S8 Relation to earlier work

S8.1 Liability-scale heritability

Lee et al. [11] provide a liability-scale conversion of binary heritability estimates that accounts for population prevalence and ascertainment. The present derivation concerns the conditional logistic projection of a per-SNP effect. At $\rho^2 = 0$, both per-SNP factors derived here reduce to λ_{marg} to first order.

S8.2 Risk-informed association testing

Zaitlen et al. [12] study informed conditioning in case-control association studies. Under the present probit model, the working-logistic factor depends on the case fraction. Section S2 derives this dependence from the sampled score equations.

S8.3 Covariate adjustment and power

Pirinen, Donnelly, and Spencer [13] showed that covariate adjustment can reduce case-control GWAS power even when the covariate is independent of genotype in the population, especially for rare disease. Informed conditioning can increase power in other settings [12]. The direction depends on prevalence, covariate effects, and SNP architecture.

Covariate adjustment defines the association being analyzed; conversion expresses it in liability units.

S8.4 Liability conversion in downstream analyses

A downstream analysis can use log-odds, observed binary, or latent-liability units. Its target determines the relevant conversion. Genetic correlation and standardized polygenic-score (PGS) prediction are invariant to a common positive factor. For direct liability effects, $A_i/\lambda_{\mathrm{marg}}$ is κ_{cond} times $A_i/\lambda_{\mathrm{cond}}$.

S9 Downstream analyses: MR, genetic variance, and polygenic scores

S9.1 Univariable Mendelian randomization

Let binary exposure X have prevalence p_X and adjusted covariates explaining ρ_X^2 of its liability variance. Outcome Y is continuous or separately calibrated to liability units. Causal interpretation requires instrument relevance, independence from exposure-outcome confounders, exclusion of other outcome pathways, and compatible adjustment targets. The population MR relationship is [14, 15, 16]

$$\beta_{G_iY} \;=\; \theta\,\beta_{G_iX}^{\mathrm{liab}} + \nu_i, \tag{S9.1}$$

where θ is the effect per unit exposure liability, $\beta_{G_iX}^{\mathrm{liab}}$ and β_{G_iY} are the population exposure and outcome associations, and ν_i describes departures from proportionality, including horizontal pleiotropy. Valid instruments give $\nu_i = 0$; sampling error enters the estimated associations.

Inverse variance-weighted (IVW) MR uses $w_i = 1/\mathrm{SE}(\widehat{\beta}_{G_iY})^2$ (main-text Eq (8)). For the liability target [17], the exposure associations use the marginal conversion for an unadjusted GWAS and the adjusted-risk conversion after collider subtraction for a direct-effect analysis.

Dividing the bias-subtracted coefficient by λ_{logit} gives the direct effect to first order. Using its approximation λ_{cond}, the noiseless MR model gives

$$\theta_{\mathrm{IVW}}^{\mathrm{marg}} \approx \frac{\theta}{\kappa_{\mathrm{cond}}(p_X, \rho_X^2)}, \qquad \kappa_{\mathrm{cond}}(p_X, \rho_X^2) = \frac{\lambda_{\mathrm{cond}}(p_X, \rho_X^2)}{\lambda_{\mathrm{marg}}(p_X)} \geq 1. \tag{S9.2}$$

For realized data and fixed weights, dividing every exposure association by κ_{cond} multiplies IVW by κ_{cond} exactly. The approximation in Eq (S9.2) concerns its relation to θ; finite-sample bias also depends on predictor error, pleiotropy, and slope estimation.

If both traits are converted, $\widehat{\theta}_{\mathrm{cond}} = (\kappa_X/\kappa_Y)\widehat{\theta}_{\mathrm{marg}}$ for fixed first-order weights. The noiseless marginal-scaled target is therefore $(\kappa_Y/\kappa_X)\theta$.

S9.2 Multivariable MR and fixed-weight rescaling

Let $X_1, \ldots, X_J$ be binary exposures with prevalences $p_1, \ldots, p_J$ and covariate-explained liability variances $\rho_1^2, \ldots, \rho_J^2$. For continuous or liability-scaled Y, assume that the SNP associations satisfy the direct-effect model

$$\beta_{G_iY} \;=\; \sum_{j=1}^{J} \theta_j \, \beta^{\text{liab}}_{G_iX_j}, \tag{S9.3}$$

where $\beta^{\text{liab}}_{G_iX_j}$ is the liability-scale SNP-exposure association and $\boldsymbol{\theta} = (\theta_1, \ldots, \theta_J)^\top$ is the vector of direct causal effects.

The corrected MVMR estimator divides each exposure's SNP-association vector by its own factor before fitting:

$$\widehat{\beta}^{\text{cond}}_{G_iX_j} = \frac{A_{ij}}{\widehat{\lambda}_{\text{cond},j}}, \qquad A_{ij} = \widehat{\beta}^{C}_{G_iX_j} - \widehat{\boldsymbol{b}}^\top_{\text{logOR},j} \widehat{\boldsymbol{\beta}}_{G_i\boldsymbol{H}_j}.$$

Let B be the matrix of marginal-scaled exposure associations, W a fixed positive definite outcome precision matrix, and $K_\kappa = \text{diag}(\kappa_{\text{cond},1}, \ldots, \kappa_{\text{cond},J})$. Conditional scaling replaces B by BK_κ^{-1}. For full column rank,

$$\widehat{\boldsymbol{\theta}}_{\text{cond}} = K_\kappa (B^\top W B)^{-1} B^\top W \boldsymbol{y} = K_\kappa \widehat{\boldsymbol{\theta}}_{\text{marg}}. \tag{S9.4}$$

The identity holds for correlated exposure columns. Under the noiseless model generated with $\lambda_{\text{cond},j}$, raw log-odds inputs instead give $D^{-1}\boldsymbol{\theta}$, where $D = \text{diag}(\lambda_{\text{cond},j})$.

Matrix identity. The fixed-weight transformation follows directly from

$$\begin{aligned}&\{(BK_\kappa^{-1})^\top W (BK_\kappa^{-1})\}^{-1} (BK_\kappa^{-1})^\top W \boldsymbol{y} \\ &= K_\kappa (B^\top W B)^{-1} B^\top W \boldsymbol{y}.\end{aligned}$$

Other MVMR estimators. For multivariable MR-Egger [18] with fixed weights, exposure rescaling changes the slopes by the same identity and leaves the intercept unchanged; outcome rescaling changes both. For robust estimators such as MVMR-cML [19], the transformation also depends on exposure SEs, constraints, penalties, and selection rules. All inputs must be rescaled consistently to assess whether the identity holds.

S9.3 Multivariable MR simulations

We generated two-exposure summary statistics with $\theta_1 = 0.4$, $\theta_2 = -0.3$, independent $\gamma_{1k}, \gamma_{2k} \sim \mathcal{N}(0, 0.06^2)$, $K = 30$, $n = 8{,}000$ per GWAS, and $R = 500$. Exposure coefficients were generated using $\lambda_{\text{cond},j}$. IVW used raw log-odds inputs or inputs divided by the marginal or conditional factor for each exposure (Table G).

Table G. MVMR estimates of joint causal effects (θ_1, θ_2) under three exposure rescalings. Two-exposure design, $\theta_1 = 0.4$, $\theta_2 = -0.3$, $K = 30$ instruments, $n = 8{,}000$ per GWAS, $R = 500$ replicates. Setting A: $p_1 = p_2 = 0.10$, $\rho_1^2 = \rho_2^2 = 0.30$. Setting B: $(p_1, \rho_1^2) = (0.10, 0.30)$, $(p_2, \rho_2^2) = (0.05, 0.10)$. Monte Carlo standard errors in parentheses.

Setting	Method	$\widehat{\theta}_1$ (MCSE)	$\widehat{\theta}_2$ (MCSE)
A	Log-OR	+0.166 (0.001)	-0.125 (0.001)
A	Marginal	+0.324 (0.001)	-0.245 (0.001)
A	Conditional	+0.372 (0.001)	-0.281 (0.001)
B	Log-OR	+0.164 (0.001)	-0.116 (0.001)
B	Marginal	+0.320 (0.001)	-0.252 (0.001)
B	Conditional	+0.368 (0.001)	-0.259 (0.001)

Conditional-scaled IVW reduced bias in both settings. The remaining underestimation of 6–14% is the regression dilution from sampling error in the exposure associations (Section S10.2). The raw log-odds slopes express effects per unit log odds of each exposure.

S9.4 Direct genetic-component variance

For independent standardized SNPs, the variance of the direct genetic component is $h^2_{\text{dir}} = \sum_i \gamma_i^2$. With LD correlation matrix R_G, it is $\boldsymbol{\gamma}^\top R_G \boldsymbol{\gamma}$. This may differ from total SNP heritability because adjustment removes mediated paths and because direct and mediated components can covary.

When the same bias-subtracted coefficients are divided by a common fixed factor and their sampling covariance by its square, a consistently noise-corrected quadratic estimate is divided by the square of that factor. Thus switching from marginal to conditional scaling multiplies the estimated direct-component variance by $\kappa_{\text{cond}}^{-2}$. Under the idealized summary-scale model $A_i = \lambda_{\text{cond}}\gamma_i + e_i$, with independent mean-zero errors of known variances, $\sum_i \{(A_i/\lambda_{\text{cond}})^2 - \text{Var}(e_i)/\lambda_{\text{cond}}^2\}$ is unbiased for h^2_{dir}. Table H shows the deterministic scaling for $h^2_{\text{dir}} = 0.20$.

Table H. Direct genetic-component variance under marginal vs conditional scaling. Deterministic expectations under the idealized summary-scale model at $p_Y = 0.10$ and true $h^2_{\text{dir}} = 0.20$.

ρ^2	κ^2_{cond}	Marginal	Conditional
0.10	1.078	0.216	0.200
0.20	1.181	0.236	0.200
0.30	1.318	0.264	0.200
0.40	1.506	0.301	0.200

Genetic correlation is invariant to consistent rescaling of covariance and variances [20] (Section S9.6).

S9.5 Polygenic-score scaling

For fixed factors and the same bias-subtracted coefficients, $\mathrm{PGS}_{\mathrm{marg}} = \sum_i (A_i/\widehat{\lambda}_{\mathrm{marg}})G_i = \kappa_{\mathrm{cond}}\mathrm{PGS}_{\mathrm{cond}}$. Regressing target liability on the score therefore gives a marginal-to-conditional slope ratio of $1/\kappa_{\mathrm{cond}}$. The positive rescaling preserves ranking and predictive R^2 (Table I).

Discovery-sample noise can attenuate either slope by the same proportion.

Table I. Polygenic-score scaling under marginal vs conditional scaling at $p_Y = 0.10$. Deterministic identities for the same score coefficients and target sample. R^2 is invariant to the constant rescaling.

ρ^2	κ_{cond}	Slope ratio (marg/cond)	R^2 ratio
0.10	1.038	0.963	1.000
0.20	1.087	0.920	1.000
0.30	1.148	0.871	1.000
0.40	1.227	0.815	1.000

Absolute-risk prediction also requires a calibrated intercept, outcome model, and target-population distribution.

S9.6 Genetic correlation

If direct genetic effects for traits A, B are multiplied by positive constants c_A, c_B, their genetic covariance becomes $c_A c_B \sigma_{g,AB}$ and their genetic variances become $c_A^2 h_A^2, c_B^2 h_B^2$. Hence

$$\frac{c_A c_B \sigma_{g,AB}}{\sqrt{c_A^2 h_A^2\, c_B^2 h_B^2}} = \frac{\sigma_{g,AB}}{\sqrt{h_A^2 h_B^2}}.$$

Common liability rescaling therefore leaves consistently calculated genetic correlation unchanged.

S10 Disease examples and public-data analyses

The T2D simulation converts exposure associations for MR with coronary artery disease (CAD); the public BMI-to-T2D analysis converts outcome associations. The AD examples comprise a hypothetical conversion grid and an LDSC estimate for the released phenotype.

S10.1 Illustrative simulation: BMI-adjusted T2D in MR with CAD

Plug-in inputs. We set T2D prevalence to $p_{\mathrm{T2D}} = 0.10$ and CAD prevalence to $p_{\mathrm{CAD}} = 0.06$ as rounded simulation design values. For context, US estimates are 11.3% for diagnosed diabetes among adults aged 20 years or older in August 2021–August 2023 [21] and 4.9% for diagnosed coronary heart disease among adults in 2025 [22]. Calibration uses prevalence for the GWAS target population and disease definition; the sample case fraction describes ascertainment. Section S7 examines prevalence sensitivity.

We set $\rho^2 = 0.30$ in the primary design and 0.20 or 0.40 in alternative designs (Table B).

Simulated data-generating process. We simulated $K = 120$ valid instruments with independent T2D effects $\gamma_k \sim \mathcal{N}(0, 0.025^2)$ and SNP-BMI effects $\beta_{G_i H,k} \sim \mathcal{N}(0, 0.02^2)$. Adjusted log-odds coefficients were $\beta^C_{G_i Y} = \lambda_{\text{cond}}(\gamma_k + b_{\text{lin}}\beta_{G_i H,k})$, with $b_{\text{lin}} = 0.50$, corresponding to opposite signs of the two confounder effects. Each design used its generating ρ^2 for conversion.

The causal effect of T2D liability on CAD liability was $\theta = 0.40$. We used $n_{\text{T2D}} = 80{,}000$, $n_{\text{BMI}} = 80{,}000$, $n_{\text{CAD}} = 100{,}000$, and $R = 100$ replicates.

Estimation and uncertainty. We generated independent Gaussian errors for the three GWAS, marginal-scaled the CAD effects and SEs, and regressed adjusted T2D effects on observed SNP-BMI associations with an intercept. After subtracting the fitted bias, we applied each exposure conversion and fitted IVW with outcome-variance weights. The intervals treat the exposure associations and fitted bias slope as fixed.

Table J. IVW MR for T2D liability → CAD liability under three exposure rescalings, with collider-bias subtraction via a fitted log-odds-scale bias slope. True liability effect $\theta = 0.40$; $R = 100$ replicates. Each group changes true ρ^2 together with its plug-in.

ρ^2	Method	Mean IVW	Mean SE	Bias (%)	Cov. (%)
0.20	Marginal	0.345	0.021	-13.8	26
0.20	Conditional	0.375	0.023	-6.3	78
0.20	Log-OR	0.177	0.011	NA	NA
0.30	Marginal	0.333	0.020	-16.9	6
0.30	Conditional	0.382	0.023	-4.5	84
0.30	Log-OR	0.171	0.010	NA	NA
0.40	Marginal	0.312	0.019	-21.9	1
0.40	Conditional	0.383	0.023	-4.1	88
0.40	Log-OR	0.160	0.010	NA	NA

NA: bias and coverage against the liability target are not applicable to log-OR exposure inputs because their units differ. Intervals condition on the fitted bias subtraction.

Conditional scaling reduced bias and improved coverage in all three designs (Table J). The conditional-to-marginal IVW ratio was exactly κ_{cond}. Section S10.2 explains the remaining bias and undercoverage.

S10.2 Regression dilution in the MR simulations

The MR simulations generate each exposure association as $\widehat{\beta}_k = \lambda\gamma_k + e_k$, with e_k independent of γ_k. After division by λ, the first-order IVW estimator with equal outcome weights converges, as the number of instruments grows, to

$$\theta D, \qquad D = \frac{\lambda^2 \operatorname{Var}(\gamma)}{\lambda^2 \operatorname{Var}(\gamma) + \operatorname{Var}(e)},$$

the classical regression-dilution factor. With independent exposure effects and independent errors, each MVMR coefficient carries its own factor D_j. In the T2D/CAD design, $\mathrm{Var}(e)$ also includes the error from subtracting the fitted bias slope, $b_\infty^2 \sigma_u^2 + (\lambda b_{\mathrm{lin}} - b_\infty)^2 \mathrm{Var}(\beta_{GH})$, where $\sigma_u^2 = 1/n_{\mathrm{BMI}}$ and $b_\infty = \lambda b_{\mathrm{lin}} \mathrm{Var}(\beta_{GH})/\{\mathrm{Var}(\beta_{GH}) + \sigma_u^2\}$ is the probability limit of the fitted regression slope. Marginal scaling multiplies the limit by a further $1/\kappa_{\mathrm{cond}}$.

Predicted and observed bias differed by at most 1.2 percentage points (Table K). This measurement-error component remains after conversion and is addressed by estimators such as MR-RAPS [23].

Table K. Predicted regression dilution and observed relative bias (%) in the MR simulations. Predictions use the design variances and the large-K limit; observed values are from Tables G and J.

		Conditional-scaled		Marginal-scaled	
Design	Coefficient	Predicted	Observed	Predicted	Observed
T2D/CAD, $\rho^2 = 0.20$	θ	−5.2	−6.3	−12.7	−13.8
T2D/CAD, $\rho^2 = 0.30$	θ	−4.7	−4.5	−17.0	−16.9
T2D/CAD, $\rho^2 = 0.40$	θ	−4.2	−4.1	−21.9	−21.9
MVMR, setting A	θ_1	−7.1	−7.0	−19.1	−19.0
MVMR, setting A	θ_2	−7.1	−6.4	−19.1	−18.5
MVMR, setting B	θ_1	−7.1	−8.0	−19.1	−19.9
MVMR, setting B	θ_2	−12.8	−13.7	−15.1	−15.9

S10.3 Hypothetical calculation motivated by Alzheimer's disease

AD prevalence and heritability depend on age, proxy phenotypes, and treatment of the *APOE* region [24]. We used hypothetical Gaussian-risk inputs to examine the conversion when the covariates explain a small fraction of liability variance.

Plug-in inputs. We set $p_{\mathrm{AD}} = 0.05$ and $\rho^2 \in \{0.04, 0.07, 0.10, 0.13\}$ (Table L). A genotype-adjusted application uses the discrete-risk calculation in Section S1.6, with the genotype coding, conditional risks, sampling design, and observational calibration for that GWAS.

Table L. Hypothetical low-variance Gaussian risk: implied conditional-prevalence correction at $p_Y = 0.05$. The marginal liability-to-logit factor is $\widehat{\lambda}_{\mathrm{marg}} = 2.171$ throughout. Computed by 200-point Gauss-Hermite quadrature.

ρ^2	$\widehat{\lambda}_{\mathrm{cond}}$	$\widehat{\kappa}_{\mathrm{cond}}$	Overstatement (%)
0.04	2.192	1.010	1.0
0.07	2.209	1.017	1.7
0.10	2.228	1.026	2.6
0.13	2.249	1.036	3.6

The low prevalence and explained variance kept κ_{cond} near one.

S10.4 Analyses of AD and BMI-adjusted T2D GWAS

We analyzed European-ancestry summary statistics using LDSC [25] via GenomicSEM [26] and IVW MR [14].

S10.4.1 AD liability heritability: Bellenguez et al. 2022

We obtained Stage 1 European-ancestry summary statistics from Bellenguez et al. 2022 [27] through the NHGRI-EBI GWAS Catalog (GCST90027158), harmonized them to HapMap3 SNPs, and applied LDSC with 1000 Genomes EUR LD scores. With sample case fraction $P = 0.141$ and population prevalence $K = 0.05$, the fit gave mean $\chi^2 = 1.27$, intercept 1.09, and liability-scale SNP heritability $\widehat{h}^2_{\text{liab}} = 0.0202$ (SE 0.0035).

The Stage 1 release combines clinical and proxy AD and is not *APOE*-adjusted. Its heritability estimate describes that released phenotype; Table L concerns a separate hypothetical adjusted-risk model.

S10.4.2 BMI to T2D Mendelian randomization: Yengo et al. 2018 and Mahajan et al. 2018

We used BMI associations from Yengo et al. [28] and BMI-adjusted T2D associations from Mahajan et al. [29], model 2. At $p_{\text{T2D}} = 0.10$ and assumed $\rho^2 = 0.30$, $\kappa_{\text{cond}} = 1.148$. The raw log-odds IVW estimate was 0.206 (SE 0.024), compared with 0.105 (0.012) after marginal conversion and 0.092 (0.011) after conditional conversion. Across assumed ρ^2 values from 0.20 to 0.64, the conditional-scaled estimate decreased from 0.097 to 0.068.

Model 2 adjusts T2D for BMI, so the converted estimate retains that adjustment and its collider contribution. A direct-effect estimate additionally requires an identified bias slope; the total BMI effect includes pathways removed by adjustment.

Variant processing and uncertainty. We selected 41,103 BMI variants at $P < 5 \times 10^{-8}$. PLINK clumping against 1000 Genomes EUR ($r^2 < 0.001$, 10 Mb) retained 521 variants, all matched to T2D by chromosome and position. Default TwoSampleMR harmonization removed 16 palindromic variants with intermediate allele frequencies, leaving 505 for analysis. Unmatched variants were excluded by design; the source studies handled participant-level missingness. IVW used $w_i = 1/\text{SE}(\widehat{\beta}_{G_iY})^2$ and $\text{SE}(\widehat{\theta}) = \{\sum_i w_i \widehat{\beta}^2_{G_iX}\}^{-1/2}$, treating exposure associations and conversion inputs as fixed.

This calculation examines the scale of a fixed IVW estimate and SE. Sample overlap, instrument strength, pleiotropy, and heterogeneity were not assessed. Variant-level associations are available from the source GWAS.

Study populations and data access. The BMI study included approximately 700,000 European-ancestry participants. The T2D study included 74,124 cases and 824,006 controls; its BMI-adjusted analysis had effective sample size 157,401 [28, 29]. The consortia document

recruitment, measurements, ethics, and consent. We used published summary statistics from GIANT (BMI), DIAGRAM (T2D model 2), and GWAS Catalog GCST90027158 (AD), with no new participant data.

S11 End-to-end validation

We estimated the bias slope and conversion factor from individual-level data at specified prevalence, then assessed direct effects, coverage, and MR against known-slope and true-factor benchmarks.

Data-generating model. Each replicate draws $K = 300$ independent biallelic variants with minor allele frequencies from $U(0.05, 0.5)$ and standardizes the genotype dosages. Of these, 100 variants affect H only, 50 affect liability only, 50 affect both with independently drawn effects, and 100 are null. Effects on H are $\mathcal{N}(0, 0.0365^2)$ and direct effects on liability $\mathcal{N}(0, 0.03^2)$ per standardized genotype. The covariate and liability are

$$H = \sum_k \beta_k G_k + 0.4\,U + \zeta, \qquad Y^* = \sum_k \gamma_k G_k + 0.4\,H + 0.5\,U + \eta,$$

with $\mathrm{Var}(\zeta) = 0.45$, $\mathrm{Var}(\eta)$ chosen for unit liability variance, and $p_Y = 0.10$. For focal variant i, define $R_H = \sum_{k \neq i} \beta_k G_k + 0.4U + \zeta$ and $R_Y = \sum_{k \neq i} \gamma_k G_k + 0.5U + \eta$. Thus R_Y is the non-focal part of $Y^* - 0.4H$. The reference bias slope and explained variance are $b_{\mathrm{lin},i} = -\,\mathrm{Cov}(R_Y, R_H)/\,\mathrm{Var}(R_H)$ and $\rho_i^2 = (0.4 - b_{\mathrm{lin},i})^2\,\mathrm{Var}(R_H)$. The common reference log-odds slope is calculated from the means of these focal-variant quantities and the first-order working-logistic factor.

Samples and association analyses. Each replicate used four independent samples: 50,000 individuals for the adjusted GWAS, drawn from the population or as 25,000 cases and 25,000 controls; 100,000 each for the GWAS of H and the outcome; and 20,000 for calibration of Y on H. The continuous outcome was $W = \theta \sum_k \gamma_k G_k + e$, with $\theta = 0.4$ and independent noise giving $\mathrm{Var}(W) = 1$. Thus $\beta_{G_i W} = \theta\gamma_i$ defines the association ratio evaluated by MR. We fitted logistic regression of Y on G_k, H by maximum likelihood and linear regressions of H and W on G_k.

Estimation. We estimated the log-odds bias slope using CWLS [7] in `indexevent` 0.2.0 [6], with all variants, and Slope-Hunter [8] in `SlopeHunter` 1.1.0, with $P < 10^{-3}$ for H and 100 bootstrap samples. Both implementations return the bias-subtracted coefficient A_i and a marginal SE incorporating estimated slope uncertainty. The known-slope analysis uses the first-order reference value described above.

For population samples, we estimated the factor either as $\lambda_{\mathrm{cond}}(p_Y, \widehat{\rho}^{\,2})$ or from the fitted Bernoulli variance in the GWAS sample (Section S6.3). The first route estimates $\widehat{\rho}^{\,2} = V/(1+V)$ by probit regression in the calibration cohort and holds $p_Y = 0.10$ fixed. Its

delta-method SE uses the fitted probit-slope variance and treats the empirical variance of H as fixed. The fitted-variance route estimates prevalence from the GWAS sample and uses 40 bootstrap samples to estimate the factor's SE. For case-control samples, we compared the population factor with $\lambda_\pi(p_Y, \widehat{\rho}^2, \pi = 0.5)$, using the specified prevalence and case fraction. Marginal conversion and the generating reference factor are included for comparison.

We used marginal SEs $\{s^2_{A_i}/\widehat{\lambda}^2 + A_i^2 s_\lambda^2/\widehat{\lambda}^4\}^{1/2}$ from Eq (S5.1), setting $\mathrm{Cov}(A_i, \widehat{\lambda}) = 0$. This covariance vanishes for independent calibration; it was omitted for the fitted-variance factor, which shares the GWAS sample with A_i. We also treated the empirical covariate variance as fixed and set cross-variant covariances from the shared slope and factor to zero. We assessed these approximations through repeated-sample coverage.

Downstream analyses and metrics. The direct genetic-component variance was estimated as $\sum_i(\widehat{\beta}_i^2 - \mathrm{SE}_i^2)$ over all 300 variants. MR used the converted associations as exposure associations and the W associations as outcome associations. We applied IVW to variants selected at $P < 5 \times 10^{-8}$ for the bias-subtracted association and profile-score MR-RAPS [23] (`mr.raps` 0.4.3; ℓ_2 loss, no over-dispersion) to all variants. IVW used outcome-variance weights and treated exposure associations as fixed. MR-RAPS used their marginal SEs, with cross-variant covariances set to zero.

Aggregate scale bias was $100\{\mathbb{E}(\sum_i \gamma_i\widehat{\beta}_i / \sum_i \gamma_i^2) - 1\}$, the mean percentage departure of the through-origin calibration slope from one. We calculated coverage separately for direct-effect, H-only, and null variants. MR bias and coverage refer to $\theta = 0.4$. Each sampling design used 200 replicates.

Results. Estimated ρ^2 matched the generating mean to three decimal places, and CWLS recovered the reference bias slope more closely than Slope-Hunter (Table M; main-text Fig 5). In population samples, the fitted-variance factor removed most scale bias and gave near-nominal coverage; the conditional-prevalence factor retained its small approximation error (Table C). In case-control samples, the sampled factor reduced scale bias and brought MR-RAPS coverage close to nominal, whereas the population factor retained about 6% scale bias.

Slope error contributes $(b - \widehat{b})\beta_{G_iH}/\lambda$ to each corrected effect. Its aggregate contribution depends on $\sum_i \gamma_i\beta_{G_iH}$, which averages toward zero under the independent-effect design. Aggregate bias can therefore be small despite slope error. IVW retained attenuation, with instruments selected from the same GWAS and exposure associations treated as fixed; MR-RAPS used all variants and their marginal SEs.

Table M. Estimation from individual-level data, with 200 replicates per design. Slope ratios appear in parentheses. Scale: aggregate scale bias (MCSE). Cov.: per-variant coverage for direct-effect and H-only variants. h^2: bias of direct genetic-component variance. MR columns give relative bias and coverage for the generating association ratio; parentheses give MCSE where shown. All entries except slope ratios are percentages. Known-slope and true-factor rows are benchmarks.

		Direct-effect estimation				MR-RAPS		IVW	
Bias slope	Factor	Scale	Cov.	Cov. H	h^2	Bias	Cov.	Bias	Cov.
Population sample ($p_Y = 0.10$)									
Known slope (1.00)	Marginal	+19.4 (0.2)	89.2	94.7	+42.8	−16.3 (0.2)	1	−21.1	0
	Conditional	+1.4 (0.2)	94.7	94.7	+3.1	−1.5 (0.3)	92	−7.2	44
	Fitted variance	+0.4 (0.2)	94.8	94.7	+1.1	−0.5 (0.3)	94	−6.2	51
	True factor	+0.4 (0.2)	94.8	94.7	+1.1	−0.5 (0.3)	94	−6.2	52
CWLS (1.01)	Marginal	+18.9 (0.2)	89.6	94.8	+41.7	−16.0 (0.2)	1	−20.9	0
	Conditional	+1.0 (0.2)	94.8	94.8	+2.3	−1.1 (0.3)	93	−7.0	45
	Fitted variance	+0.0 (0.2)	94.8	94.8	+0.3	−0.1 (0.3)	94	−6.0	52
	True factor	+0.0 (0.2)	94.8	94.8	+0.3	−0.1 (0.3)	96	−6.0	54
Slope-Hunter (1.11)	Marginal	+19.4 (0.2)	89.6	95.0	+42.4	−16.2 (0.2)	1	−21.2	0
	Conditional	+1.5 (0.2)	94.8	95.0	+2.8	−1.4 (0.3)	93	−7.3	44
	Fitted variance	+0.5 (0.2)	94.9	95.0	+0.8	−0.4 (0.3)	94	−6.4	51
	True factor	+0.5 (0.2)	94.8	95.0	+0.8	−0.4 (0.3)	94	−6.4	50
Case-control sample ($p_Y = 0.10$, $\pi = 0.5$)									
Known slope (1.00)	Marginal	+25.2 (0.1)	75.4	95.0	+56.7	−20.1 (0.2)	0	−21.9	0
	Conditional	+6.3 (0.1)	93.0	95.0	+13.0	−6.0 (0.2)	48	−8.0	16
	Sampled factor	+0.4 (0.1)	94.9	95.0	+0.8	−0.4 (0.2)	94	−2.6	82
	True factor	+0.3 (0.1)	94.9	95.0	+0.7	−0.4 (0.2)	94	−2.6	83
CWLS (0.99)	Marginal	+24.8 (0.1)	76.6	95.4	+55.7	−19.9 (0.2)	0	−21.8	0
	Conditional	+6.0 (0.1)	93.4	95.4	+12.3	−5.6 (0.2)	51	−7.9	18
	Sampled factor	+0.1 (0.1)	94.9	95.4	+0.1	−0.1 (0.2)	94	−2.4	82
	True factor	+0.1 (0.1)	95.0	95.4	+0.1	−0.1 (0.2)	94	−2.4	84
Slope-Hunter (1.07)	Marginal	+25.2 (0.1)	75.6	94.9	+56.8	−20.2 (0.2)	0	−21.9	0
	Conditional	+6.3 (0.1)	92.9	94.9	+13.1	−6.0 (0.2)	48	−8.1	14
	Sampled factor	+0.4 (0.1)	94.7	94.9	+0.8	−0.4 (0.2)	93	−2.7	80
	True factor	+0.4 (0.1)	94.8	94.9	+0.8	−0.4 (0.2)	94	−2.6	80

S12 Implementation and reproducibility

Algorithm A implements the population Gaussian conditional-prevalence approximation. For a population GWAS, the fitted-variance factor in Section S6.3 gives the exact first-order conversion. For case-control GWAS, use the sampled factor and corresponding bias slope (Section S2). Apply the procedure separately to each exposure in MVMR and transform all uncertainty inputs consistently (Section S9.2).

Algorithm A Conditional-prevalence correction for covariate-adjusted binary GWAS

Require: Adjusted log-odds GWAS estimates $\{(\widehat{\beta}^{C}_{G_iY}, \mathrm{SE}^{C}_{i})\}_{i=1}^{K}$; SNP-covariate associations $\{\widehat{\boldsymbol{\beta}}_{G_i\boldsymbol{H}}\}_{i=1}^{K}$ with covariance estimates; population prevalence $0 < p_Y < 1$; covariate-explained liability variance $0 \leq \rho^2 < 1$. Estimated inputs require a joint sampling covariance, including calibration and bias-slope uncertainty.

Ensure: Conditional-scaled liability-scale estimates $\{(\widehat{\beta}^{\mathrm{cond}}_{G_iY^*}, \mathrm{SE}(\widehat{\beta}^{\mathrm{cond}}_{G_iY^*}))\}_{i=1}^{K}$.

1: Set

$$t_Y = \Phi^{-1}(1 - p_Y),\ \tau = \sqrt{1 - \rho^2}.$$

2: Compute

$$Q = \mathbb{E}_{M \sim N(0,\rho^2)} \left[\Phi \left(\frac{M - t_Y}{\tau} \right) \left\{ 1 - \Phi \left(\frac{M - t_Y}{\tau} \right) \right\} \right],$$

using Gauss-Hermite quadrature, and set

$$\lambda_{\mathrm{cond}} = \frac{\phi(t_Y)}{Q}, \qquad \kappa_{\mathrm{cond}} = \frac{\lambda_{\mathrm{cond}}}{\phi(t_Y)/\{p_Y(1 - p_Y)\}}.$$

3: Obtain a consistent estimate of the covariate-bias vector $\widehat{\boldsymbol{b}}_{\mathrm{logOR}}$ and its joint covariance with the SNP associations. The estimator's identification, dimension, and sampling assumptions must hold for the adjusted binary GWAS (Section S3).

4: **for** $i = 1, \ldots, K$ **do**

5: Bias-correct the adjusted log-odds estimate:

$$A_i = \widehat{\beta}^{C}_{G_iY} - \widehat{\boldsymbol{b}}^{\top}_{\mathrm{logOR}} \widehat{\boldsymbol{\beta}}_{G_i\boldsymbol{H}}.$$

6: Convert to the corrected liability scale:

$$\widehat{\beta}^{\mathrm{cond}}_{G_iY^*} = \frac{A_i}{\lambda_{\mathrm{cond}}}.$$

7: Compute $\mathrm{SE}(\widehat{\beta}^{\mathrm{cond}}_{G_iY^*})$ by the joint delta method in Eq (S5.1), including nuisance covariance where applicable.

8: **end for**

9: **return**

$$\left\{ \left(\widehat{\beta}^{\mathrm{cond}}_{G_iY^*}, \mathrm{SE}(\widehat{\beta}^{\mathrm{cond}}_{G_iY^*}) \right) \right\}_{i=1}^{K},\ \kappa_{\mathrm{cond}}.$$

References

1. Prentice RL, Pyke R. Logistic disease incidence models and case-control studies. Biometrika. 1979;66(3):403-11. doi:10.1093/biomet/66.3.403.
2. Fithian W, Hastie T. Local case-control sampling: Efficient subsampling in imbalanced data sets. Ann Stat. 2014;42(5):1693-724. doi:10.1214/14-AOS1220.
3. Zhou W, Nielsen JB, Fritsche LG, Dey R, Gabrielsen ME, Wolford BN, et al. Efficiently controlling for case-control imbalance and sample relatedness in large-scale genetic association studies. Nat Genet. 2018;50(9):1335-41. doi:10.1038/s41588-018-0184-y.
4. Mbatchou J, Barnard L, Backman J, Marcketta A, Kosmicki JA, Ziyatdinov A, et al. Computationally efficient whole-genome regression for quantitative and binary traits. Nat Genet. 2021;53(7):1097-103. doi:10.1038/s41588-021-00870-7.
5. Firth D. Bias reduction of maximum likelihood estimates. Biometrika. 1993;80(1):27-38. doi:10.1093/biomet/80.1.27.
6. Dudbridge F, Allen RJ, Sheehan NA, Schmidt AF, Lee JC, Jenkins RG, et al. Adjustment for index event bias in genome-wide association studies of subsequent events. Nat Commun. 2019;10:1561. doi:10.1038/s41467-019-09381-w.
7. Cai S, Hartley A, Mahmoud O, Tilling K, Dudbridge F. Adjusting for collider bias in genetic association studies using instrumental variable methods. Genet Epidemiol. 2022;46:303-16. doi:10.1002/gepi.22455.
8. Mahmoud O, Dudbridge F, Davey Smith G, Munafò M, Tilling K. A robust method for collider bias correction in conditional genome-wide association studies. Nat Commun. 2022;13:619. doi:10.1038/s41467-022-28119-9.
9. Wang P, Lin Z, Xue H, Pan W. Collider bias correction for multiple covariates in GWAS using robust multivariable Mendelian randomization. PLoS Genet. 2024;20(4):e1011246. doi:10.1371/journal.pgen.1011246.
10. Berisa T, Pickrell JK. Approximately independent linkage disequilibrium blocks in human populations. Bioinformatics. 2016;32(2):283-5. doi:10.1093/bioinformatics/btv546.
11. Lee SH, Wray NR, Goddard ME, Visscher PM. Estimating missing heritability for disease from genome-wide association studies. Am J Hum Genet. 2011;88(3):294-305. doi:10.1016/j.ajhg.2011.02.002.
12. Zaitlen N, Lindström S, Pasaniuc B, Cornelis M, Genovese G, Pollack S, et al. Informed conditioning on clinical covariates increases power in case-control association studies. PLoS Genet. 2012;8(11):e1003032. doi:10.1371/journal.pgen.1003032.
13. Pirinen M, Donnelly P, Spencer CCA. Including known covariates can reduce power to detect genetic effects in case-control studies. Nat Genet. 2012;44(8):848-51. doi:10.1038/ng.2346.
14. Burgess S, Butterworth A, Thompson SG. Mendelian randomization analysis with multiple genetic variants using summarized data. Genet Epidemiol. 2013;37(7):658-65. doi:10.1002/gepi.21758.
15. Bowden J, Davey Smith G, Burgess S. Mendelian randomization with invalid instruments: effect estimation and bias detection through Egger regression. Int J Epidemiol. 2015;44(2):512-25. doi:10.1093/ije/dyv080.
16. Bowden J, Davey Smith G, Haycock PC, Burgess S. Consistent estimation in Mendelian randomization with some invalid instruments using a weighted median estimator. Genet Epidemiol. 2016;40(4):304-14. doi:10.1002/gepi.21965.

17. Burgess S, Labrecque JA. Mendelian randomization with a binary exposure variable: interpretation and presentation of causal estimates. Eur J Epidemiol. 2018;33:947-52. doi:10.1007/s10654-018-0424-6.

18. Rees JMB, Wood AM, Burgess S. Extending the MR-Egger method for multivariable Mendelian randomization to correct for both measured and unmeasured pleiotropy. Stat Med. 2017;36(29):4705-18. doi:10.1002/sim.7492.

19. Lin Z, Xue H, Pan W. Robust multivariable Mendelian randomization based on constrained maximum likelihood. Am J Hum Genet. 2023;110(4):592-605. doi:10.1016/j.ajhg.2023.02.014.

20. Bulik-Sullivan B, Finucane HK, Anttila V, Gusev A, Day FR, Loh PR, et al. An atlas of genetic correlations across human diseases and traits. Nat Genet. 2015;47(11):1236-41. doi:10.1038/ng.3406.

21. Gwira JA, Fryar CD, Gu Q. Prevalence of total, diagnosed, and undiagnosed diabetes in adults: United States, August 2021–August 2023. National Center for Health Statistics; 2024. 516. doi:10.15620/cdc/165794.

22. National Center for Health Statistics. FastStats: Heart disease. Centers for Disease Control and Prevention; 2026. National Health Interview Survey 2025 estimate; accessed September 28, 2026. Available from: `https://www.cdc.gov/nchs/fastats/heart-disease.htm`.

23. Zhao Q, Wang J, Hemani G, Bowden J, Small DS. Statistical inference in two-sample summary-data Mendelian randomization using robust adjusted profile score. Ann Stat. 2020;48(3):1742-69. doi:10.1214/19-AOS1866.

24. Liu S, Bush WS, Akinyemi RO, Byrd GS, Caban-Holt AM, Rajabli F, et al. Alzheimer disease is (sometimes) highly heritable: Drivers of variation in heritability estimates for binary traits, a systematic review. PLoS Genet. 2025;21(9):e1011701. doi:10.1371/journal.pgen.1011701.

25. Bulik-Sullivan BK, Loh PR, Finucane HK, Ripke S, Yang J, Patterson N, et al. LD Score regression distinguishes confounding from polygenicity in genome-wide association studies. Nat Genet. 2015;47(3):291-5. doi:10.1038/ng.3211.

26. Grotzinger AD, Rhemtulla M, de Vlaming R, Ritchie SJ, Mallard TT, Hill WD, et al. Genomic structural equation modelling provides insights into the multivariate genetic architecture of complex traits. Nat Hum Behav. 2019;3(5):513-25. doi:10.1038/s41562-019-0566-x.

27. Bellenguez C, Küçükali F, Jansen IE, Kleineidam L, Moreno-Grau S, Amin N, et al. New insights into the genetic etiology of Alzheimer's disease and related dementias. Nat Genet. 2022;54(4):412-36. doi:10.1038/s41588-022-01024-z.

28. Yengo L, Sidorenko J, Kemper KE, Zheng Z, Wood AR, Weedon MN, et al. Meta-analysis of genome-wide association studies for height and body mass index in ∼700,000 individuals of European ancestry. Hum Mol Genet. 2018;27(20):3641-9. doi:10.1093/hmg/ddy271.

29. Mahajan A, Taliun D, Thurner M, Robertson NR, Torres JM, Rayner NW, et al. Fine-mapping type 2 diabetes loci to single-variant resolution using high-density imputation and islet-specific epigenome maps. Nat Genet. 2018;50(11):1505-13. doi:10.1038/s41588-018-0241-6.
