# Supplementary material for: Conditional-prevalence correction for liability-scale analyses of covariate-adjusted binary GWAS
Source: PLoS Genet. 2026 Oct 30;22(10):e1012348. doi: 10.1371/journal.pgen.1012348 (PMC13722303; doi:10.1371/journal.pgen.1012348)
Supplement: Supplementary file 2 [file pgen.1012348.s003.pdf]

S1 Checklist. STROBE-MR reporting checklist

PGENETICS-D-26-00549 | Siyi Chen | 14 October 2026

Reporting locations for the methodological study, simulations, and public-data sensitivity calculation. Page numbers refer to the clean main manuscript (21 pages) and S1 Text (26 pages). Item descriptions are abbreviated; the numbering follows the STROBE-MR checklist.

Item / topic	Location	Relevant text and reporting information
1 Title and abstract	Main, pp. 1-2	The title identifies covariate-adjusted binary GWAS. The abstract defines collider bias, liability, and direct effects, states the shared conversion factor under the Gaussian model and small genetic effects, and explains why prevalence alone can misstate effect magnitude. It summarizes the population-sample results and the extension to case-control sampling.
2 Background	Main 1, p. 3-4	The Introduction develops the BMI-adjusted T2D example, distinguishes collider bias from mediation, defines liability units, and compares prior bias-correction, heritability, and association-testing work. It states the contribution and explains why variation in risk across covariates matters for effect magnitude.
3 Objectives	Main 2.1, p. 5-7	The target is the structural direct genetic effect on liability. The manuscript distinguishes this target from the mediated and total effects and evaluates the conversion after an identified collider component is subtracted.
4 Design and data	Main 3.2, p. 13; Main 3.5, p. 16; S10.4.2, p. 20-21	We combine analytic derivations, deterministic quadrature, individual-level simulations, summary-statistic MR simulations, an end-to-end individual-level simulation, and public-data sensitivity calculations. Each design and its role are described separately.
4a Setting	S10.4.2, p. 20-21	BMI statistics are from Yengo et al. (2018), and BMI-adjusted T2D statistics are from Mahajan et al. (2018), model 2. Both source studies concern European ancestry. The manuscript cites the source reports for recruitment and measurement details.
4b Samples and size	S4, p. 9-10; S9.3, p. 15; S11, p. 21-22; S10.4.2, p. 20-21	Individual-level designs report sample sizes and replicate counts; downstream designs report instrument counts. Each end-to-end replicate used a GWAS sample of 50,000, covariate and outcome GWAS of 100,000 each, and a calibration cohort of 20,000. The BMI source study included about 700,000 participants. The T2D BMI-adjusted analysis had effective sample size 157,401. No new participant recruitment or empirical power calculation was conducted.
4c Variant selection	S10.4.2, p. 20-21	BMI instruments used P < 5 x 10^-8 and European LD clumping at r-squared < 0.001 within 10 Mb. Clumping retained 521 matched variants; harmonization excluded 16 palindromic variants, leaving 505 instruments.
4d Trait assessment	Main 2.1, p. 5-7; S10.4.2, p. 20-21	The model defines binary disease by a threshold on continuous liability. The public illustration uses BMI and BMI-adjusted T2D associations. The originating GWAS reports describe phenotype ascertainment, transformations, covariates, and measurement.

Item / topic	Location	Relevant text and reporting information
4e Ethics and consent	S10.4.2, p. 20-21	We used published summary statistics with no new participant data. The originating consortia document recruitment, measurements, ethics, and consent.
5 IV assumptions	S9.1, p. 14-15	S9.1 states relevance, independence from exposure-outcome confounders, and exclusion restriction. Exposure and outcome associations must describe compatible pathways after adjustment. Main 2.1 and Fig 1 give the separate structural assumptions for the conversion.
6a Variables and units	Main 2.5, p. 11; S9.1, p. 14-15	The model standardizes focal genetic variation and liability. The direct liability coefficient, adjusted log-odds coefficient, conversion factor, and first-order IVW weights are defined. Binary-exposure MR targets a liability effect; the public example rescales the T2D outcome association.
6b Weights	Main 2.5, p. 11; S10.4.2, p. 20-21	The first-order IVW weight is the inverse variance of the SNP-outcome association. S10.4.2 gives the weighted regression-through-origin calculation and its fixed-input SE. No participant-level allele score is used for the public MR calculation.
6c Estimators	S9.1, p. 14-15; S9.2, p. 15-16; S10.4.2, p. 20-21	The supplement gives IVW and fixed-weight multivariable rescaling equations and describes the simulated estimators. BMI-adjusted T2D associations and BMI exposure associations represent different adjustment targets; the public results are presented as numerical sensitivity calculations.
6d Missing data	S10.4.2, p. 20-21	Unmatched variants were excluded by design. All 521 clumped variants matched the outcome before harmonization, which removed 16 palindromic variants with intermediate allele frequencies. The source studies handled participant-level missingness.
6e Multiple testing	S10.4.2, p. 20-21; S4, p. 9-10	The public instrument-selection threshold is P < 5 x 10^-8. The study reports conversion bias, coverage, and sensitivity rather than a set of new causal discoveries. No additional multiplicity correction was applied to those descriptive comparisons.
7 Assumption checks	Main 2.1, p. 5-7; S3, p. 8; S10.4.2, p. 20-21	The structural assumptions are stated and imposed in the relevant simulations. Bias-slope identification is discussed separately. Instrument-strength, pleiotropy, heterogeneity, and sample-overlap diagnostics were not computed for the public-data example.
8 Sensitivity methods	S1.6, p. 6; S2, p. 7; S6, p. 11-13; S7, p. 12	Analyses examine population versus case-control sampling, conditional-variance approximation error, non-Gaussian risk distributions, and assumed calibration. The fixed-model sensitivity analysis varies the conversion input while holding the generating model fixed.
9a Software	S12, p. 23-24	Verified numerical checks used R 4.5.1, statmod 1.5.2, mvtnorm 1.4-2, and MASS 7.3-66. The cbcGWAS R package, including analysis functions and simulation code, is available on GitHub. Package versions were not recorded for the public-GWAS analyses.

Item / topic	Location	Relevant text and reporting information
9b Registration	Author statement	This study was not preregistered.
10a Analysis flow	S10.4.2, p. 20-21	Clumping retained 521 outcome-matched variants; harmonization excluded 16, leaving 505 instruments. Source GWAS participant selection and exclusions are described in the original publications.
10b Sample description	S10.4.2, p. 20-21	The source-study ancestry, reported sample sizes, phenotype sources, and adjusted release are identified. Participant-level means, SDs, and disease counts for contributing cohorts are given in the source reports.
10c Source-study variation	S10.4.2, p. 20-21	Study-specific samples and adjustments are described in the cited source publications. The public-data calculation does not analyze differences between contributing cohorts.
10d(i) Cross-sample comparability	S9.1, p. 14-15; S10.4.2, p. 20-21	S9.1 requires exposure and outcome associations to represent compatible pathways. Both public source GWAS concern European ancestry. Transportability and the consequences of the T2D covariate adjustment remain assumptions for a causal interpretation.
10d(ii) Sample overlap	S10.4.2, p. 20-21	The calculation examines the scale of a fixed IVW estimate and SE. Sample overlap, instrument strength, pleiotropy, and heterogeneity were not assessed.
11a Variant associations	S10.4.2, p. 20-21; S1 Data; submission-form Data Availability statement	S10.4.2 reports the aggregate estimates, calibration inputs, and instrument count. Variant-level associations are available from the original GWAS data sources linked in the submission-form Data Availability statement and S1 Data.
11b Effects and uncertainty	Main 3.4, p. 15-16; S9.3, p. 15-16; S10.4.2, p. 20-21	Simulation tables report bias, Monte Carlo uncertainty, SE summaries, and coverage. The public IVW estimate is 0.206 (SE 0.024); marginal and conditional-prevalence conversions give 0.105 (SE 0.012) and 0.092 (SE 0.011), respectively, for the stated fixed inputs.
11c Absolute risk	Main 2.5, p. 11; S10.4.2, p. 20-21	No absolute-risk effect is estimated. The methods concern log-odds and latent-liability units, and the public illustration reports a scale conversion. These quantities are not presented as clinical risk differences.
11d Main result plots	Main 3.1, p. 12-17	Fig 2 shows conversion factors and approximation error. Fig 3 shows individual-level bias and coverage. Fig 4 shows calibration sensitivity. Fig 5 shows the end-to-end validation. Numerical summaries and MR results are reported in S1 Text.
12a Assumption results	S3, p. 8; S10.4.2, p. 20-21	The paper reports the consequences of specified structural and calibration assumptions. The public-data calculation is interpreted as sensitivity to scale, not as evidence that the IV assumptions hold.
12b Diagnostic statistics	S10.4.2, p. 20-21	F, Q, and I-squared statistics were not computed for the public-data MR calculation. Instrument selection used genome-wide significance and LD clumping.

Item / topic	Location	Relevant text and reporting information
13a Robustness results	Main 3.3, p. 14-15; Main 3.5, p. 16-17; S6, p. 11-13	The results quantify approximation error, sensitivity to assumed explained liability variance, and sampling effects. The end-to-end simulation estimates the bias slope with published methods and the conversion factor from the data, and reports aggregate scale bias, per-variant coverage, and MR bias and coverage. Cohort-based calibration uses the specified prevalence. The attenuation and undercoverage remaining in the MR simulations match regression dilution from sampling error in the exposure associations. Calibration values for the public examples are assumed rather than empirically estimated.
13b Additional analyses	S9, p. 14-18; S10, p. 17-21	Additional analyses cover multivariable MR, direct genetic-component variance, polygenic scores, T2D-to-CAD simulation, regression dilution, end-to-end validation, and AD sensitivity. The numerical comparisons are reported in S1 Text, with their underlying values in S1 Data.
13c Direction of causation	Main 2.1, p. 5-7; S10.4.2, p. 20-21	The model specifies causal direction, and Fig 1 is explicitly hypothetical. No empirical reverse-direction or Steiger test is reported for the public BMI/T2D calculation.
13d Other causal designs	S10.4.2, p. 20-21	No new observational-regression or randomized-trial effect is estimated for comparison. The public calculation compares one IVW estimate under different conversion factors.
13e Further plots	Main 3.3, p. 14; S9.3, p. 15; S10.4.2, p. 20-21	Fig 4 and supplementary tables report additional numerical comparisons. No public-data MR scatter, funnel, or leave-one-out plot is supplied because a diagnostic reanalysis of individual variant associations was not performed.
14 Principal findings	Main 4, p. 18-19	The Discussion explains how variation in risk across covariates affects liability conversion, relates the result to bias correction and downstream genetic analyses, and describes calibration and reporting in population and case-control studies. It distinguishes aggregate scale accuracy from individual-effect accuracy and scale bias from regression dilution.
15 Limitations	Main 4, p. 18-19	The paper discusses the local Gaussian expansion, approximation error, sampling by disease status, calibration transportability, bias-slope identification, and incomplete public-data correction. The simulation used a calibration cohort drawn from the GWAS population. S11 specifies the marginal SE approximation and its omitted covariance terms.
16a Interpretation	Main 4, p. 18-19	Claims are tied to the derived local conversion and evaluated numerical designs. Common rescaling changes effect units and fixed-input intervals while preserving the corresponding Wald statistic. Numerical rescaling alone does not establish a corrected causal effect.

Item / topic	Location	Relevant text and reporting information
16b Mechanistic interpretation	Main 2.1, p. 5-7; S9.1, p. 14-15	The direct, mediated, and total effects are distinguished. The binary-exposure MR target concerns exposure liability. The study does not equate a lifelong genetic-liability effect with a specific environmental or clinical intervention.
16c Clinical implications	Main 4, p. 18-19	The work informs interpretation and reporting of adjusted genetic associations. No treatment recommendation or new clinical effect is inferred from the illustrative disease calculations.
17 Generalizability	Main 4, p. 18-19; S6, p. 11-12	Application requires compatible prevalence, measurement, ancestry, age distribution, calibration, and sampling. The Gaussian local model and common-factor identities do not establish validity for arbitrary non-Gaussian or heterogeneously selected studies.
18 Funding	Submission form	The journal financial disclosure is: "The author(s) received no specific funding for this work." Funding is entered in the journal submission form, following PLOS instructions. No external funder role is reported.
19 Data and code	S12, p. 23-24; S10.4.2, p. 20-21	S1 Text contains the formatted numerical tables; S1 Data provides the underlying numerical values. The cbcGWAS R package provides analysis functions and simulation code. The Data Availability statement gives full links to BMI data from Yengo et al. (2018), BMI-adjusted T2D data from Mahajan et al. (2018), model 2, and AD data from Bellenguez et al. (2022), accession GCST90027158.
20 Competing interests	Submission form; Author Comments	The authors have declared that no competing interests exist. This journal wording is consistent with the author-confirmed declaration.

Checklist structure: STROBE-MR / EQUATOR Network, CC BY 3.0.
https://www.strobe-mr.org/download/strobe-mr-checklist-fillable/
